# Correcting geometric distortions in stereoscopic 3D imaging

**DOI:** 10.1371/journal.pone.0205032

**Published:** 2018-10-08

**Authors:** Zhongpai Gao, Alex Hwang, Guangtao Zhai, Eli Peli

**Affiliations:** 1 Schepens Eye Research Institute, Massachusetts Eye and Ear, Department of Ophthalmology, Harvard Medical School, Boston, MA, United States of America; 2 Institute of Image Communication and Information Processing, Shanghai Jiao Tong University, Shanghai, China; Nanjing University of Information Science and Technology, CHINA

## Abstract

Motion in a distorted virtual 3D space may cause visually induced motion sickness. Geometric distortions in stereoscopic 3D can result from mismatches among image capture, display, and viewing parameters. Three pairs of potential mismatches are considered, including 1) camera separation vs. eye separation, 2) camera field of view (FOV) vs. screen FOV, and 3) camera convergence distance (i.e., distance from the cameras to the point where the convergence axes intersect) vs. screen distance from the observer. The effect of the viewer’s head positions (i.e., head lateral offset from the screen center) is also considered. The geometric model is expressed as a function of camera convergence distance, the ratios of the three parameter-pairs, and the offset of the head position. We analyze the impacts of these five variables separately and their interactions on geometric distortions. This model facilitates insights into the various distortions and leads to methods whereby the user can minimize geometric distortions caused by some parameter-pair mismatches through adjusting of other parameter pairs. For example, in postproduction, viewers can correct for a mismatch between camera separation and eye separation by adjusting their distance from the real screen and changing the effective camera convergence distance.

## Introduction

Stereoscopic 3D (S3D) is being used for virtual/augmented reality, scientific visualization, medical imaging, 3D movies, and gaming. The ultimate goal of S3D systems is to convey the real world or virtually constructed 3D world veridically to the viewer. However, it is often the case that various S3D capture, display, and viewing parameters are mismatched [1]. This may introduce geometric distortions for the viewer [2–4]. Such space distortions may degrade the quality of stereoscopic presentation [5] and user’s performances on size/distance estimations for virtual interactions, which are known to be beneficial in S3D [6]. Geometric space distortions also interfere with the viewer’s perception of self-motion. When they are inconsistent with the familiar real-world motion perception, they may cause visually induced motion sickness (VIMS) [3]. Therefore, understanding the sources of such geometric distortions with the aim of correcting or minimizing effects should be the starting point of improving overall quality of the S3D presentation.

The S3D imaging chain includes capturing the original 3D world (real or virtual) by two cameras, and displaying the S3D content dichoptically on dichoptic screens, and finally viewing the S3D contents by users. The capture and display parameters of the S3D imaging chain can be grouped into the corresponding pairs: 1) camera separation vs. eye separation (interpupillary distance, IPD), 2) camera field of view (FOV) vs. screen FOV, 3) camera convergence distance vs. screen distance. Camera convergence distance is the distance from the midpoint between the cameras to the point where the camera convergence axes intersect. The viewer initiated viewing parameters such as translational offset can be expressed as the distance from the designated (optimal) head position.

Woods et al. [4] provided a transfer function from the real (or virtual) world to the S3D world. Using this model, various geometric distortions were analyzed, such as depth plane curvature (i.e., objects are bent away from the viewer in periphery, see also [3]), depth non-linearity (i.e., depth differences in the reconstructed world do not match with the corresponding depth differences in the original world), and shearing distortion (i.e., objects appear sheared toward the viewer’s head position) [7].

The geometric model developed by Woods, et al. [4] demonstrates how individual parameters in the S3D imaging chain may affect the final presentation to the viewer. However, since the parameters involved in the S3D imaging chain were not explicitly grouped into corresponding pairs, it is hard to intuitively understand the interaction among the parameter pairs. In Woods, et al. [4], to demonstrate the effect of the various display parameters, the other parameters were assigned to fixed default values. Camera and eye separation were assigned 75mm and 65mm, respectively, whereas camera FOV was assigned 50° or 52° and screen FOV was assigned 17° (calculated from 1*m* screen distance and 30*cm* screen width). Since geometric distortions may result from a combination of multiple mismatches (due to mismatches of multiple paired parameters), it is unclear whether the distortion patterns found through such analysis were caused entirely by a solo effect of the examined parameter pair, or the combined effect with other default parameter mismatches. For instance, when demonstrating the effect of camera separation, the simulated distortions were confounded by the mismatch between camera FOV and screen FOV.

Our geometric model is expressed as a function of the ratios of the three parameter-pairs: 1) camera separation vs. eye separation, 2) camera field of view (FOV) vs. screen FOV, and 3) camera convergence distance vs. screen distance from the observer. The geometric distortions as a function of each parameter ratio can be studied independently by assuming the other pairs are perfectly matched. Yet, one can then consider the interactions among the parameter pairs by changing more than one ratio at a time. Using a model expressed in terms of ratios of paired corresponding parameters facilitates intuition about the effects and leads to a better understanding of the relationship between the parameter pairs. The effect of viewer’s suboptimal head positions (i.e., the head is offset from the screen center) is also discussed.

For real screen displays (e.g., smartphone, monitor, TV, and movie theater), where the screen size is fixed, changing the screen distance changes the screen FOV. The user’s eye separation varies with the user. In the case of pre-produced content, such as watching S3D movies, the contents capture parameters are set during the initial capture and postproduction phases (e.g., convergence distance may be adjustable by horizontally translating the displayed images [8]), but they are typically not allowed to be adjusted by the viewer.

The simplest approach to correct the geometric distortions is to match the capture, display, and viewing systems. However, the user’s eye separation and camera separation are fixed and they may be different from each viewer. Our model shows that it is possible to adjust other controllable parameter pairs to compensate for the distortions caused by the mismatch between eye separation and camera separation. Specifically, we propose a method to remove the geometric distortions during S3D viewing by adjusting the screen distance and camera convergence distance (i.e., horizontally shifting left and right captured views).

The existence of depth distortions in S3D is well known and some distortions have been named. Masaoka et al. [9] and Yamanoue’s [10] geometric models were used to analyze commonly reported S3D perceptual size and depth distortions, known as the puppet-theater effect [11] and cardboard effect [12]. The puppet-theater effect is caused by size/scale discrepancies between objects in the real world and those reconstructed in S3D. For example, when reconstructed objects in the foreground are relatively smaller than objects in the background (while accounting for the perceived distance), the viewer perceives the objects in the foreground to be relatively smaller as if they are figures in a puppet theater. The cardboard effect is caused by non-linearly compressed depth, such that farther objects appear to be more compressed in depth than closer objects and thus they may be perceived flatter, and in extreme, as a cardboard cutout of a picture of the objects. The opposites of these two effects are also possible, where the objects reconstructed in S3D appear larger relative to the background (giant effect) or farther objects are expanded non-linearly in depth (referred to here as an expansion effect). We use our model to analyze the mismatches of parameter pairs that lead to the various depth distortions effects.

We assume here that: 1) there is no viewer’s head rotation relative to the screen; 2) stereoscopic images are captured by parallel-axis method (with sensor shift) and are displayed on a flat screen. The camera image plane (the image plane perpendicular to the camera axes) and screen image plane (the image plane on which the screen is located) are matched. Note that, when the viewer’s head is rotated with respect to the displayed images, or when stereoscopic images are captured by convergence-axis (toe-in) method but displayed on a flat screen, additional geometric distortions may be introduced [2, 3]. Moreover, as pointed by [2], such distortions are accompanied by vertical disparities, resulting in no intersection between two projection lines from the left and right eyes to a pair of onscreen points. Thus, one cannot use ray-intersection geometric models to predict geometric distortions in such situations. Therefore, head orientation mismatch and image plane mismatch that also involve vertical disparities require a special handling and analysis and are outside the scope of the current paper.

## The process of S3D imaging

In S3D viewing, captured objects at the convergence distance are displayed with zero disparity and perceived as if they are at the screen distance. The objects captured in front of the convergence distance (displayed in crossed disparity) are perceived as if they are in front of the screen, while objects captured behind the convergence distance (displayed in uncrossed disparity) are perceived behind the screen.

**Fig 1:**
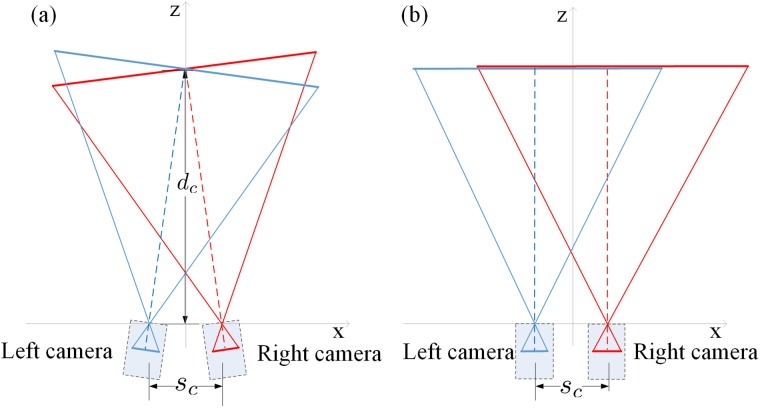
Two common capture configurations for stereoscopic systems (top view). *s*_*c*_ is the cameras separation. *d*_*c*_ is the camera convergence distance. (a) Converging cameras, also called *toe-in*. (b) Parallel cameras.

S3D content acquisition (capture) involves a pair of cameras that are horizontally separated. For simplicity of derivation, we ignore lens distortions by assuming pinhole cameras, which are commonly implemented in virtual world computer graphic rendering. For stereo image capture, two capture methods are commonly used: converging-cameras method and parallel-cameras method, as shown in Fig 1. In the converging-cameras method, also called *toe-in*, (Fig 1a), the axes of the two cameras converge. The distance from the midpoint between the two cameras to the convergence point is called camera convergence distance (*d*_*c*_). Images captured in this way presented on parallel displays (or a single stereo display) result in a severe geometric distortion due to the projection difference. Thus this system is rarely used. In the parallel-cameras method (Fig 1b), the axes of two cameras are parallel, making *d*_*c*_ to be infinite.

The parallel-cameras method captures all the objects in the scene in crossed disparities and therefore, they are all perceived to be in front of the display screen. The parallel-cameras method thus compresses the full scene depth into the distance between the viewer and the screen. This is an example of an extreme mismatch between corresponding parameters (pair) resulting in a large distortion of depth. In addition, the parallel-cameras acquisition often results in large crossed disparities for close objects, which may exceed the viewer’s binocular fusion range. To avoid this severe distortion and fusion limitation, the camera convergence distance has to be shortened, preferably to match with the display viewing distance.

**Fig 2:**
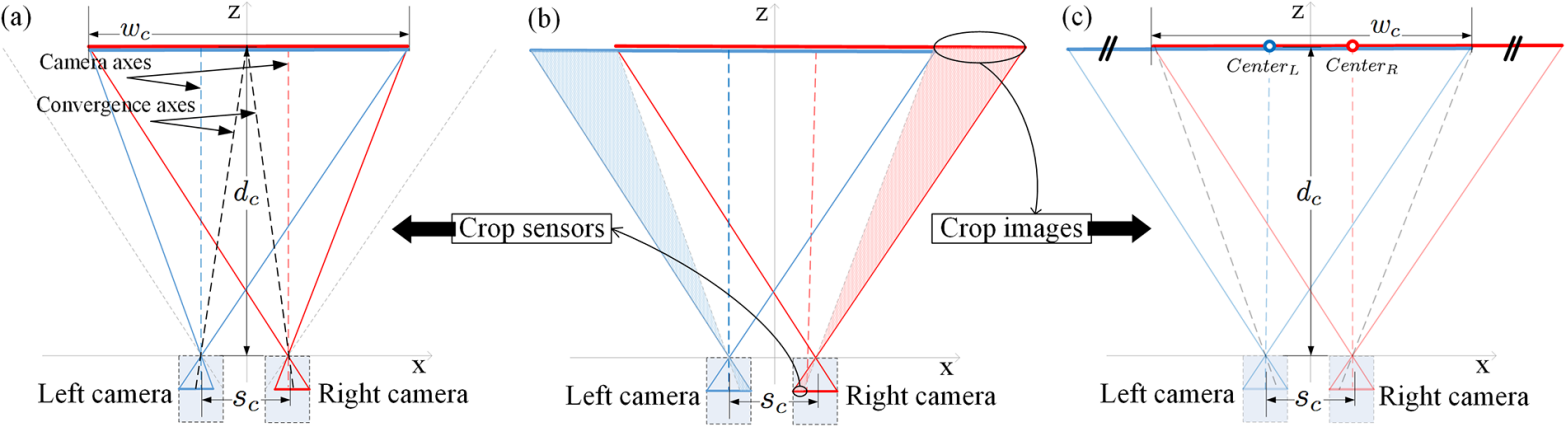
Two methods of controlling the convergence distance in the parallel-cameras capture. (a) Cropping sensor method. The right side of the left sensor and left side of the right sensor are cut out compared to the full-size sensors in (b). This method can also be considered as shifting the left sensor to the left and right sensor to the right compared to the smaller-size sensors in Fig 1b, so it is also referred to as ‘*sensor-shift*’. The convergence distance (*d*_*c*_) is the intersection of the convergence axes (projection line from the retained sensor center to the camera aperture). (c) Cropping image method. The left side of the left captured image and right side of the right captured image are cut out compared to the full-size images in (b). This method is also referred to as ‘*image-shift*’. This is because when without cropping the sensors or captured images, the centers of displayed images (*Center*_*L*_ and *Center*_*R*_) are aligned to the screen center. The left and right displayed images need to be shifted back to the left and right, respectively, and then the non-overlapping image sections are cropped. The cameras and projection lines in (c) are presented in low contrast to indicate that the cropping image method is used in postproduction. The two methods in (a) and (c) thus produce equivalent outcomes. Blue and red projection lines indicate the FOVs of the left and right cameras, respectively.

In real-world parallel-cameras capture, the convergence distance can be adjusted by horizontally shifting each camera’s image sensor outward (i.e., left camera sensor to the left and right camera sensor to the right) compared to Fig 1b. This is referred as ‘*sensor-shift*’ and is equivalent to only utilizing the outer part of the full image sensors in Fig 2a. In computer graphic capture, the convergence distance can be adjusted by creating asymmetric camera frusta for the two virtual cameras (Fig 2a) to achieve *off-axis projection* [13], which results in the same effects as ‘*shift-sensor*’ in real-world capture. Another method is cropping image method used in postproduction. The left side and right side of the left and right captured images are cut out as shown in Fig 2c). When the images are displayed on the screen without cropping sensors or images, the centers of captured images (*Cneter*_*L*_ and *Center*_*R*_ in Fig 2c) are aligned to the screen center (i.e., shift left image to the right and right image to the left), resulting in infinite convergence distance (referring back to the capture process). One can reduce the convergence distance by horizontally shifting the displayed images back (left image to the left and right image to the right) in the postproduction [8, 14], then cropping the non-overlapped area. The cropping sensor method in Fig 2a and cropping image method in Fig 2c are equivalent and achieve the same result.

The convergence distance means the distance at which the convergence axes (called optical axes in [4]) of the two cameras intersect. The convergence axis is the projection line passing through the pinhole aperture to the center of the image sensor (either real or virtual).

**Fig 3:**
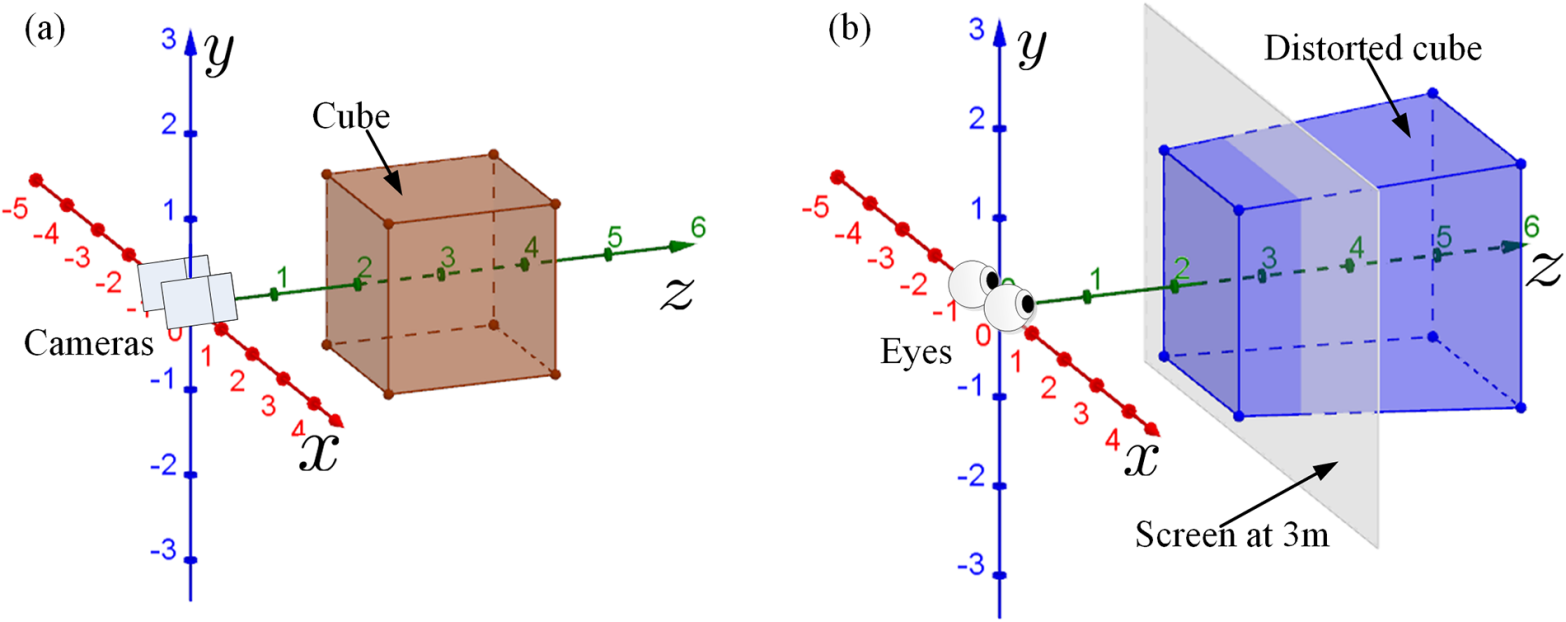
Sample scene configuration of 3D simulations. (a) The brown cube is in the original world. The left and right cameras are at: *C*_*l*_ = [−*s*_*c*_/2, 0, 0]^⊺^ and *C*_*r*_ = [*s*_*c*_/2, 0, 0]^⊺^, respectively, where *s*_*c*_ is the camera separation. (b) The blue object is an example of the distorted cube in the reconstructed world corresponding to the brown cube in (a). The gray plane is the display screen at the screen distance. The left and right eyes are *E*_*l*_ = [−*s*_*e*_/2, 0, 0]^⊺^ and *E*_*r*_ = [*s*_*e*_/2, 0, 0]^⊺^, respectively, where *s*_*e*_ is the eye separation. In subsequence figures, the capture coordinates and display coordinates are superimposed to aid the visualization of distortions.

The variables used in our geometric models are defined in Table 1. A left-handed Cartesian coordinate system *xyz* is used for both capture and display. For image capture, shown in Fig 3a, the origin is the midpoint between the left and right (real/virtual) cameras. Camera positions are at the pinhole apertures. The *x*-axis represents inter-camera direction (i.e., the horizontal axis). The *z*-axis represents the direction where the cameras are pointed (i.e., the depth axis). The *y*-axis is orthogonal to the *xz*-plane (i.e., the vertical axis). For image display, we assume that the viewer’s head is primarily positioned in front of the center of display images and does not rotate relative to the displayed images. As shown in Fig 3b, the origin is in front of the display center and at the midpoint between the left and right viewing eyes. Eye positions are assumed to be at the entrance pupils. The *x*-axis represents intraocular direction to the right (i.e., the horizontal axis). The *z*-axis represents the direction from the origin to the display (i.e., the depth axis). The *y*-axis is orthogonal to the *xz*-plane (i.e., the vertical axis).

**Table 1:** Variables for geometric models.

*s*_*c*_	camera separation (meters)
*s*_*e*_	eye separation (meters)
*k*_*s*_ = *s*_*e*_/*s*_*c*_	ratio of eye separation to camera separation
*d*_*c*_	camera convergence distance (meters)
*d*_*s*_	screen distance from the viewer (meters)
*k*_*d*_ = *d*_*s*_/*d*_*c*_	ratio of screen distance to camera convergence distance
*α*_*ch*_	horizontal camera field of view (FOV) (degrees)
*α*_*sh*_	horizontal screen FOV (degrees)
*w*_*s*_ = 2*d*_*s*_ tan(*α*_*sh*_/2)	screen width
*w*_*c*_ = 2*d*_*c*_ tan(*α*_*ch*_/2)	camera frustum width at convergence distance *d*_*c*_
*k*_*w*_ = *w*_*s*_/*w*_*c*_	ratio of screen width to camera frustum width at *d*_*c*_
$k_{f}=\frac{2d_{s}\;\text{tan}\left(\alpha_{sh}/2\right)}{2d_{s}\;\text{tan}\left(\alpha_{ch}/2\right)}$	ratio of screen width to camera frustum width at *d*_*s*_,
$\;\;\;\;=\frac{\text{tan}(\alpha_{sh}/2)}{\text{tan}(\alpha_{ch}/2)}=\frac{k_{w}}{k_{d}}$	*k*_*f*_ represents the ratio of screen FOV to camera FOV in linear scale
*O* = [*X*_*o*_, *Y*_*o*_, *Z*_*o*_]^⊺^	coordinates of a point in the original world
*P* = [*X*_*p*_, *Y*_*p*_, *Z*_*p*_]^⊺^	coordinates of the corresponding point of *O* in the reconstructed world
*T* = [*T*_*x*_, *T*_*y*_, *T*_*z*_]^⊺^	offset of head position relative to the screen center
*C*_*l*_, *C*_*r*_	positions of left and right cameras, respectively
*E*_*l*_, *E*_*r*_	positions of left and right eyes, respectively
*S*_*l*_, *S*_*r*_	positions of left and right onscreen points relative to *O*, respectively

The brown cube in Fig 3a is an example object in the original (virtual) world captured for display in S3D. The blue object in Fig 3b is the reconstructed (perceived) object in the S3D world. In the following illustrations, the brown cube center is at [0, 0, 3*m*]^⊺^ in the original world, and the length of the side of the cube is 2*m*. Any difference between the corresponding features of the brown cube (Fig 3a) and blue hexahedron (reconstructed cube) (Fig 3b) represents geometric distortions introduced by the parameter mismatches among the capture, display, and viewing processes. In subsequent simulations, the captured cube and reconstructed cube are superimposed on a single coordinate system to emphasize the distortions/differences between the *original world* and *reconstructed world*.

## S3D spatial distortion analysis

**Fig 4:**
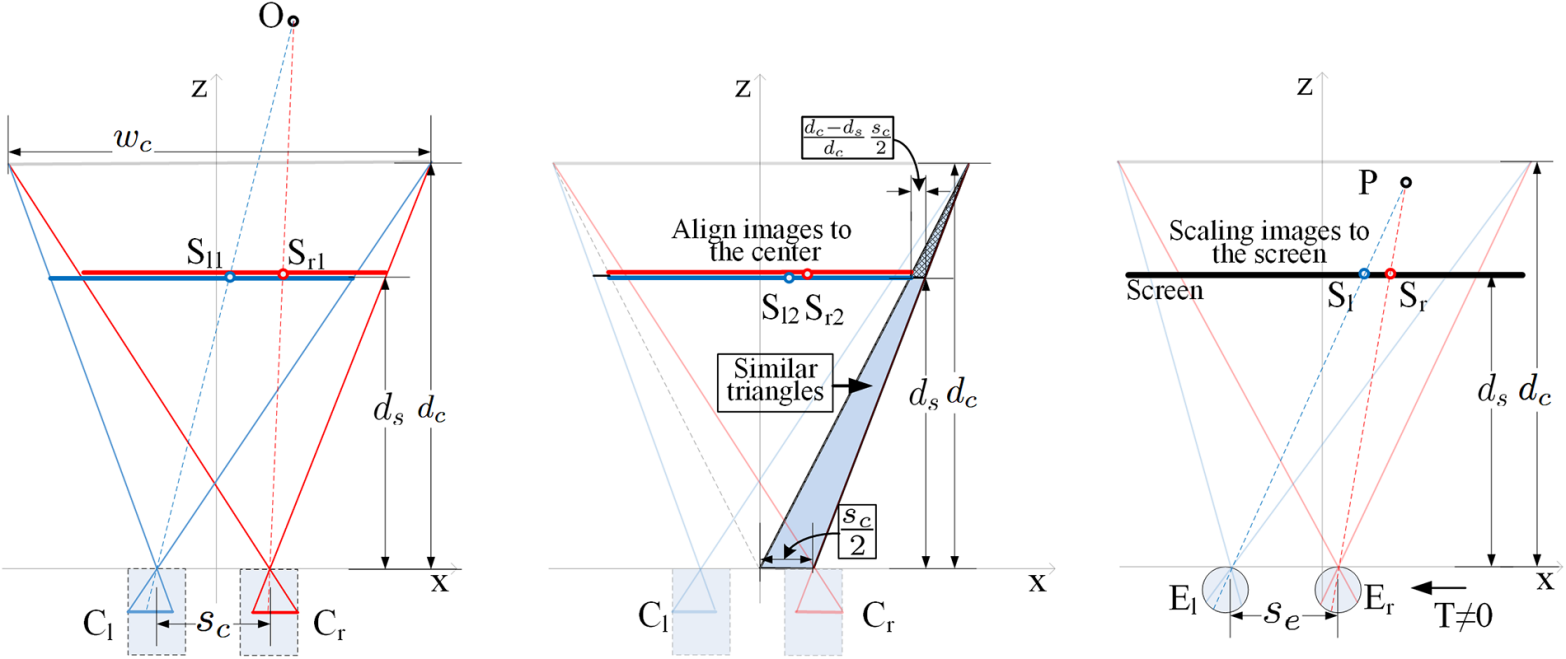
A geometric model for S3D scene capture and display. (a) Stereoscopic images are captured by parallel cameras configuration with convergence distance *d*_*c*_. The lines from the left and right pinhole cameras (*C*_*l*_ and *C*_*r*_) to object (point *O*) in the original world intersect with a plane at the screen distance *d*_*s*_ at *S*_*l*1_ and *S*_*r*1_. (b) When the captured images are displayed on a single screen display, and the centers of the captured images align at the center of the screen, the left and right images will be displaced by ($\frac{d_{c}-d_{s}}{d_{c}}s_{c}/2$), which can be calculated from the two similar triangles of different height (blue). The points *S*_*l*1_ and *S*_*r*1_ on the screen distance are displayed at *S*_*l*2_ and *S*_*r*2_. (c) The captured realigned images are scaled to fill the display screen. The points *S*_*l*2_ and *S*_*r*2_ at the screen distance are changed to *S*_*l*_ and *S*_*r*_ on the screen. Viewers will see the left and right points (*S*_*l*_ and *S*_*r*_) on the screen through the left and right eyes (*E*_*l*_ and *E*_*r*_), respectively. The intersection point *P* of the two lines from each eye (*E*_*l*_ and *E*_*r*_) to the corresponding onscreen point (*S*_*l*_ and *S*_*r*_) is the expected perceived position of *O* from (a) displayed to the observer. Note that when *d*_*s*_ < *d*_*c*_ the point *P* is displayed closer to the observer in the reconstructed world than in the original world.

In this paper, the original world is captured by parallel-cameras with the shifted sensor method and then displayed on a real flat screen. Spatial distortions are introduced by the offset of the head position (*T*) and the mismatches between the three parameter pairs: 1) camera separation vs. eye separation, 2) camera frustum width at convergence distance vs. screen width, 3) camera convergence distance vs. screen distance. Note that since changing the screen distance affects the screen FOV for real screen displays, we replace the ratio of the angular pair of camera FOV and screen FOV (*k*_*f*_) with the ratio of the linear pair of camera frustum width at the convergence distance (i.e., *w*_*c*_ in Fig 2a) and screen width (*k*_*w*_). This enables us to analyze the effects of screen size and distance separately. Fig 4 shows the diagrams used for the derivation of the geometric model. The transfer function from the original world coordinates to the reconstructed world coordinates can be expressed as

$$P=[\begin{array}{c} X_{p}\\ Y_{p}\\ Z_{p} \end{array}]=\frac{k_{s}k_{w}d_{c}}{Z_{o}\left(k_{s}-k_{w}\right)+k_{w}d_{c}}[\;\begin{array}{c} X_{o}\\ Y_{o}\\ Z_{o}\frac{k_{d}}{k_{w}} \end{array}\;]+\frac{k_{w}\left(d_{c}-Z_{o}\right)}{Z_{o}\left(k_{s}-k_{w}\right)+k_{w}d_{c}}[\;\begin{array}{c} T_{x}\\ T_{y}\\ T_{z}\frac{k_{d}}{k_{w}} \end{array}\;],$$ (1)

where *O* = [*X*_*o*_, *Y*_*o*_, *Z*_*o*_]^⊺^ is a point in the original world, *P* = [*X*_*p*_, *Y*_*p*_, *Z*_*p*_]^⊺^ is the corresponding point to be perceived in the reconstructed world; *T* = [*T*_*x*_, *T*_*y*_, *T*_*z*_]^⊺^ is the offset of head position from the origin; $k_{s}=\frac{s_{e}}{s_{c}}$ is the ratio of eye separation to camera separation, $k_{w}=\frac{w_{s}}{w_{c}}$ is the ratio of screen width to camera frustum width at convergence distance, and $k_{d}=\frac{d_{s}}{d_{c}}$ is the ratio of screen distance to camera convergence distance. See the Appendix for derivation. Note that the transfer function for *x* and *y* components are equal, while they are different for the *z* component. This indicates that the amount of distortions along the horizontal and vertical diections (along the *x* and *y* axises) are the same, while the amount distortion along the depth direction (along the *z* axis) may be different.

The transfer function is a function of the camera convergence distance, *d*_*c*_, three ratios (*k*_*s*_, *k*_*w*_, *k*_*d*_) representing three types of mismatches, and the head position offset, *T*. When the three paired parameters are matched and without head translation, i.e., *k*_*s*_ = 1, *k*_*w*_ = 1, *k*_*d*_ = 1, *T* = [0, 0, 0]^⊺^, Eq (1) can be simplified as

$$P={\left[X_{p},Y_{p},Z_{p}\right]}^{⊺}={\left[X_{o},Y_{o},Z_{o}\right]}^{⊺}=O.$$ (2)

This indicates that if the corresponding parameter pairs for capture and display systems are matched, an orthoscopic display condition will be achieved, and any point in the original world will be reconstructed exactly where it should be during the S3D viewing.

Since the viewer cannot see objects behind the viewer, depth coordinates in the reconstructed world *Z*_*p*_ should be always positive (*Z*_*p*_ > 0). When *k*_*s*_ < *k*_*w*_ and $Z_{o}>\frac{k_{w}d_{c}}{k_{w}-k_{s}}$ (i.e., for depth farther than $\frac{k_{w}d_{c}}{k_{w}-k_{s}}$), *Z*_*p*_ is negative. In this case, two projection lines (from the two eyes to the two onscreen points) intersect behind the viewer because the (uncrossed) disparity of onscreen points is larger than the viewer’s IPD. Depending on how large the angular disparity is, the viewer may perceive the object at a far distance or fail to fuse (having double vision). Note that, since $k_{w}=\frac{w_{s}}{w_{c}}=\frac{w_{s}/2}{d_{c}\;\text{tan}\left(\alpha_{ch}/2\right)}$ is independent of the screen distance (*d*_*s*_), changing the screen distance does not change the linear screen disparity (Eq (27)).

In following sections, we discuss the effect of each parameter-pair mismatch and head translations in isolation, assuming that other paired parameters are matched.

### Effect of different eye separations

This analysis assumes that the screen distance and camera convergence distance are the same (*k*_*d*_ = 1), the screen width and camera frustum width at convergence distance are the same (*k*_*f*_ = 1), and camera convergence distance is constant (i.e., *d*_*c*_ = 3*m*), while head position is at the optimal position (*T* = [0, 0, 0]^⊺^). Only camera separation and eye separation are mismatched due to individual users’ IPD variations. In this condition, the transfer function (1) is simplified as follows:

$$P=[\begin{array}{c} X_{p}\\ Y_{p}\\ Z_{p} \end{array}]=\frac{k_{s}d_{c}}{Z_{o}\left(k_{s}-1\right)+d_{c}}[\begin{array}{c} X_{o}\\ Y_{o}\\ Z_{o} \end{array}].$$ (3)

If *k*_*s*_ < 1 (i.e., the viewer’s IPD is smaller than camera separation), object depths *Z*_*o*_ should be smaller than $\frac{d_{c}}{1-k_{s}}$, otherwise the point *P* falls behind the observer, as discussed above.

**Fig 5:**
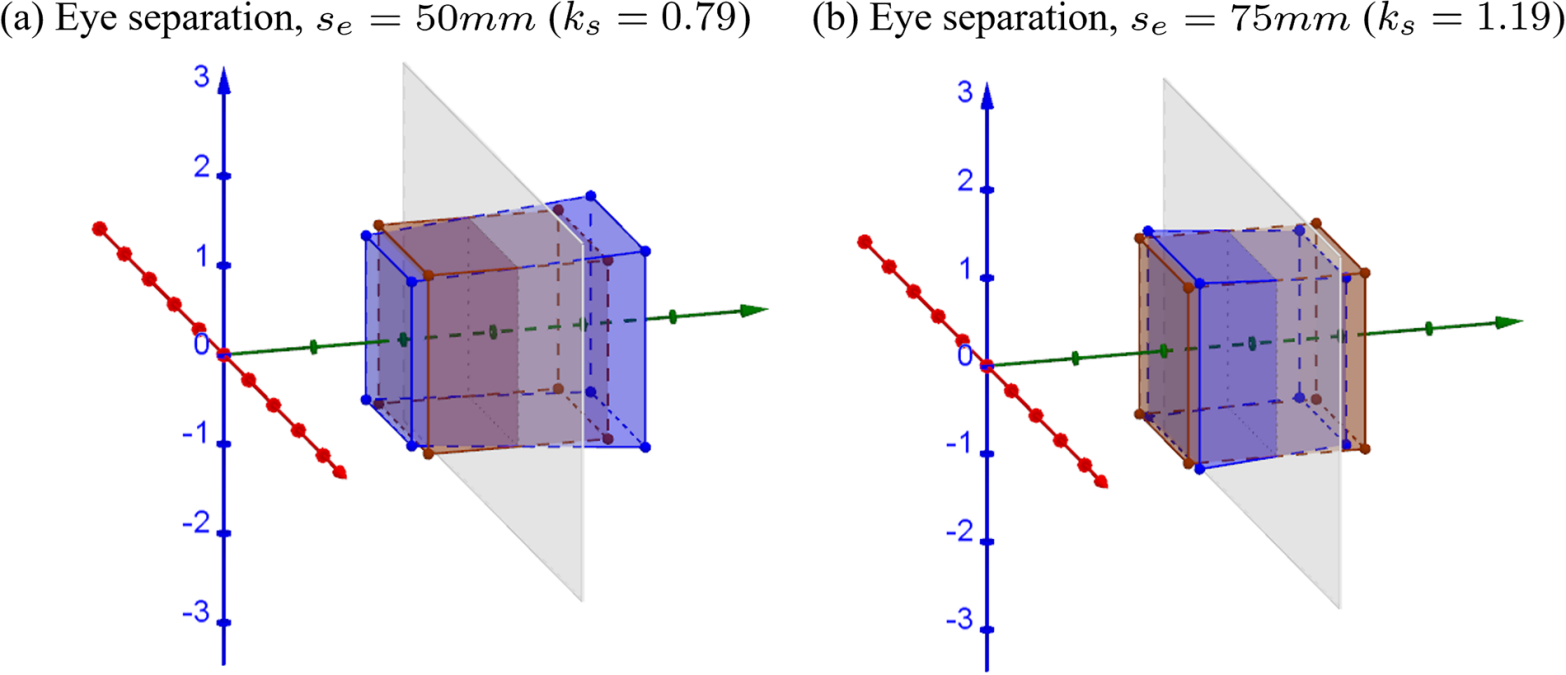
3D simulations of the effect of mismatch between the camera and eye separations. (a) IPD = 50*mm* and (b) IPD = 75*mm*. The camera separation is assumed to be *s*_*c*_ = 63*mm* and convergence distance to be *d*_*c*_ = 3*m*. The brown cube is an orthoscopic representation of the 2*m* cube centered at [0, 0, 3*m*]^⊺^ in the original world. The gray plane represents the display screen located at 3*m* distance.

Fig 5 shows simulations of a cube captured with camera separation (*s*_*c*_) of 63*mm*, which is a recommended camera separation for S3D movie making [15], while eye separation is that of a small child, 50*mm* (*k*_*s*_ = 0.79 < 1, Fig 5a), and an adult with widely-separated-eyes, 75*mm* (*k*_*s*_ = 1.19 > 1, Fig 5b), respectively. The vast majority of adults have IPDs in the range of [50*mm*, 75*mm*], where the mean value of adult IPD is around 63*mm* [16].

When eye separation is smaller than camera separation (*k*_*s*_ < 1), the reconstructed cube (i.e., blue hexahedron) appears expanded in depth (Fig 5a). The portion in front of the screen is narrower while the portion behind the screen is wider than what it is supposed to be in the orthoscopic condition. When eye separation is larger than camera separation (*k*_*s*_ > 1), the reconstructed cube appears compressed (Fig 5b), where the portion in front of the screen becomes wider and the portion behind the screen becomes narrower. The results in Fig 5 are different from the results in [2] (see Fig 1A and 1I in the Appendix of [2]). In our simulations, onscreen points stay on the screen when eye separation and camera separation are mismatched. The explanation for the discrepancy is presented in the discussion.

**Fig 6:**
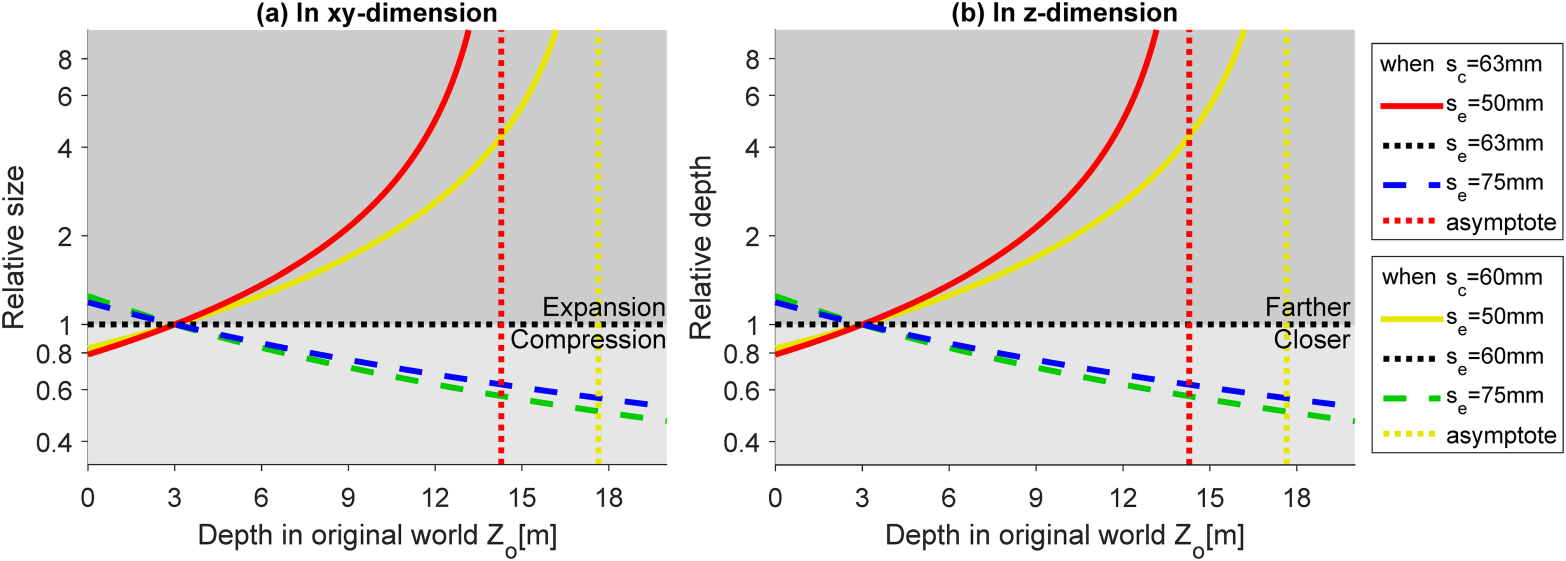
Effects of the mismatch between the camera and eye separations. (a) and (b) show the relative size (in *xy*-dimension) and depth (in *z*-dimension) of the reconstructed world as a function of the depth in the original world. The horizontal black dotted lines (relative size = 1 in (a) or relative depth = 1 in (b)) represent exact reconstruction of the original world (orthoscopic reconstruction). For given camera separation *s*_*c*_ = 63*mm*, the red solid lines and blue dashed lines represent eye separation smaller (*s*_*e*_ = 50*mm*, *k*_*s*_ < 1) and larger (*s*_*e*_ = 75*mm*, *k*_*s*_ > 1), respectively. For the condition that camera separation is reduced (*s*_*c*_ = 60*mm*), the yellow solid line and green dashed line represent smaller and larger eye separations, respectively. The red and yellow dotted vertical lines are the asymptotes of the red and yellow curves.

Fig 6 shows the change in relative size along *x* and *y*-axis (Fig 6a) and depth along *z*-axis (Fig 6b) between the original world and reconstructed world, as functions of the depth *Z*_*o*_ in the original world. The relative size and depth can be expressed as $\frac{X_{p}}{X_{o}}=\frac{Y_{p}}{Y_{o}}=\frac{k_{s}d_{c}}{Z_{o}\left(k_{s}-1\right)+d_{c}}$ and $\frac{Z_{p}}{Z_{o}}=\frac{k_{s}d_{c}}{Z_{o}\left(k_{s}-1\right)+d_{c}}$, respectively. Note that, the equations and the plots for *X*, *Y*, and *Z* dimensions are the same, resulting in the same change in all dimensions. This is because when changing the eye separation, the intersection of the two projections lines (from left and right eyes to left and right onscreen points) will always lie on the line passing through the origin (middle of two eyes) and the center of the onscreen points. The ratios of the *x*, *y*, and *z* components of any two points on a line passing through the origin are the same. In these plots, the black dotted horizontal lines represent the orthoscopic condition (i.e., a reconstruction without geometric distortion) when eye separation and camera separation are matched (in addition to other matched parameters). The area below the black horizontal line represents compression and above the line represents expansion.

Fig 6a represents relative size change (i.e., *xy*-dimension) along the depth direction. When eye separation is smaller than camera separation (*k*_*s*_ < 1), reconstructed objects in front of the screen appear smaller and objects behind the screen appear larger in size. The amount of compression and expansion increases non-linearly as objects are farther from the screen location (red/yellow solid line in Fig 6a). When eye separation is larger than camera separation (*k*_*s*_ > 1), objects in front of the screen appear expanded and objects behind the screen appear compressed (blue/green dashed line in Fig 6a). The effect is more dramatic in a smaller IPD condition than a larger IPD condition. A smaller camera separation (e.g., *s*_*c*_ = 60*mm*) decreases the distortions and allows a larger asymptotic limit (yellow lines in Fig 6a), yet it has a relatively small increase in distortions for larger IPD users (green dashed line in Fig 6a).

Fig 6b represents relative depth change (i.e., *z*-dimension) along the depth direction. The area below and above the horizontal line represents objects being closer and farther than where they are in the original world, respectively (Fig 6b). When eye separation is smaller than camera separation (*k*_*s*_ < 1), reconstructed objects in front of and behind the screen appear closer and farther, respectively. The amount of depth distortion increases non-linearly as objects are farther from the screen location (red/yellow solid line in Fig 6b). When eye separation is larger than camera separation (*k*_*s*_ > 1), objects in front of the screen appear farther and objects behind the screen appear closer (blue/green dashed line in Fig 6b).

The red/yellow dotted lines are the asymptotes (i.e., $Z_{o}=\frac{d_{c}}{1-k_{s}}$) of the red/yellow curves when eye separation is smaller than camera separation. Objects at the depth of the asymptote (and beyond), onscreen uncrossed disparities become larger than the viewer’s IPD. In this case, viewers may not be able to fuse them even if they try to fixate on those objects and perceive double vision. Note that in real-world condition, when a viewer gaze on a near object, a farther object becomes double, but when the viewer gazes on the farther objects, the farther objects will be fused (becomes single) and the near object becomes double. However, in the reconstructed world, the objects beyond the asymptote distance cannot be fused even if the viewer gazes on it. Thus, this distance represents a practical limit on the distance of the original world that can be reconstructed veridically in S3D with unmatched eyes/cameras separation parameters (see further discussion of this property below at section ‘*Avoid large screen disparity*’).

### Effect of different screen sizes

Here we assume that only screen width and camera frustum width at the convergence distance are mismatched (i.e., *k*_*s*_ = 1, *k*_*d*_ = 1, and *T* = [0, 0, 0]^⊺^) and camera convergence distance is constant (i.e., *d*_*c*_ = 3*m*). Under this assumption, the ratio between screen FOV and camera FOV (*k*_*f*_) becomes the same as the ratio between screen width and camera frustum width, i.e., *k*_*f*_ = *k*_*w*_/*k*_*d*_ = *k*_*w*_. The transfer function (1) can be simplified as follows:

$$P=[\begin{array}{c} X_{p}\\ Y_{p}\\ Z_{p} \end{array}]=\frac{k_{w}d_{c}}{Z_{o}\left(1-k_{w}\right)+k_{w}d_{c}}[\begin{array}{c} X_{o}\\ Y_{o}\\ Z_{o}/k_{w} \end{array}],$$ (4)

If *k*_*w*_ > 1 (screen width is larger than camera frustum width at convergence distance), the depth should be $Z_{o}<\frac{k_{w}d_{c}}{k_{w}-1}$, for farther *Z*_*o*_ the point *P* falls behind the observer.

**Fig 7:**
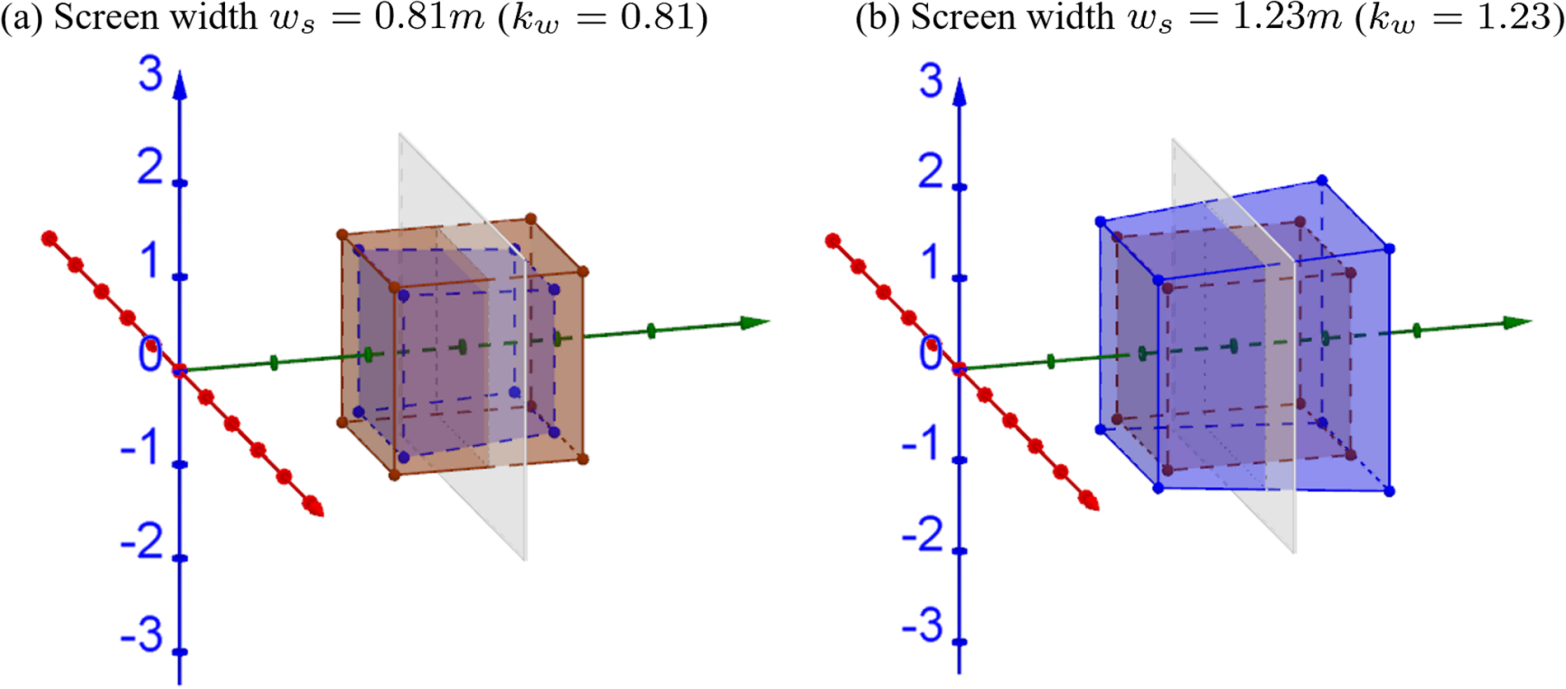
3D simulations of the effects of different screen sizes. The camera frustum width at convergence distance is assumed to be *w*_*c*_ = 1*m*. (a) With smaller screen size, scaling factor *k*_*w*_ = 0.81 and (b) with larger screen size, *k*_*w*_ = 1.23. The camera convergence distance is assumed to be fixed *d*_*c*_ = 3*m*. The brown cube and gray plane are the same as in Fig 5.

Fig 7 shows examples of the 3D simulations when screen and camera frustum widths are mismatched. When screen width is smaller than camera frustum width (*k*_*w*_ = 2^−0.3^ = 0.81 < 1), the cube appears smaller, and the farther portion is more compressed than the closer portion, as shown in Fig 7a. When screen width is larger than camera frustum width (*k*_*w*_ = 2^0.3^ = 1.23 > 1), the cube appears bigger, and the farther portion is more expanded than the closer portion, as shown in Fig 7b. Since we assume camera convergence distance and screen distance are matched, the reconstructed cube stays centered at the screen distance.

**Fig 8:**
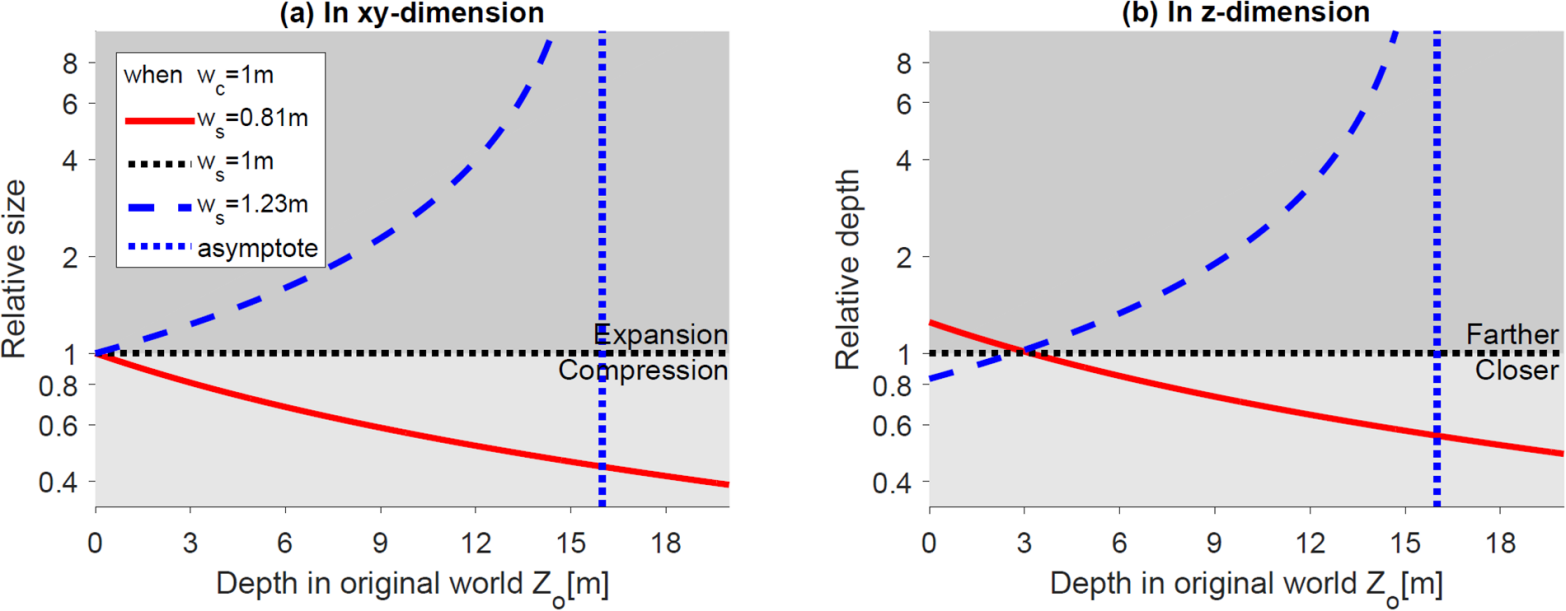
Effect of different screen sizes (i.e., using larger or smaller screen to view the content). (a) and (b) show the relative size (in *xy*-dimension) and depth (in *z*-dimension), respectively of the reconstructed world along the *z*-axis. The blue dotted vertical lines are the asymptotes of the blue curves. The horizontal black lines (relative size = 1 or depth = 1) represent no geometric distortion in the reconstructed world.

Fig 8 shows that the relative size and depth change compared to the orthoscopic condition in *xy*-dimension and *z*-dimension. The relative size in *xy*-dimension and relative depth in *z*-dimension can be expressed as $\frac{X_{p}}{X_{o}}=\frac{Y_{p}}{Y_{o}}=\frac{k_{w}d_{c}}{Z_{o}\left(1-k_{w}\right)+k_{w}d_{c}}$ and $\frac{Z_{p}}{Z_{o}}=\frac{d_{c}}{Z_{o}\left(1-k_{w}\right)+k_{w}d_{c}}$, respectively. When screen width is smaller (red solid lines) or larger (blue dashed lines) than camera frustum width, the relative size becomes smaller or larger than 1, suggesting the reconstructed world appears compressed or expanded, respectively (Fig 8a). In terms of depth, when screen width is smaller (red solid lines) or larger (blue dashed lines), than camera frustum width, the virtually constructed world behind the screen will suffer from progressive compression, while the world in front of the screen will suffer from expansion, respectively (Fig 8b). Note that objects located at the screen distance are not largely affected in terms of depth distortion, but are still affected by size distortion. The blue dotted lines are the asymptotes (i.e., $Z_{o}=\frac{k_{w}d_{c}}{k_{w}-1}$) of the blue curves when screen width is larger than camera frustum width. Again, the viewer may not be able to fuse objects farther than the asymptote and perceive double vision.

### Effect of changing screen distance

This analysis assumes that only camera convergence distance and screen distance are mismatched (i.e., *k*_*s*_ = 1, *k*_*w*_ = 1, and *T* = [0, 0, 0]^⊺^) where camera convergence distance is constant (i.e., *d*_*c*_ = 3*m*). The transfer function (1) can be simplified as follows:

$$P={\left[X_{p},Y_{p},Z_{p}\right]}^{⊺}={\left[X_{o},Y_{o},k_{d}Z_{o}\right]}^{⊺}.$$ (5)

Eq (5) shows that changing screen distance affects the depth (in *z*-dimension) but does not affect the size (in *xy*-dimensions).

**Fig 9:**
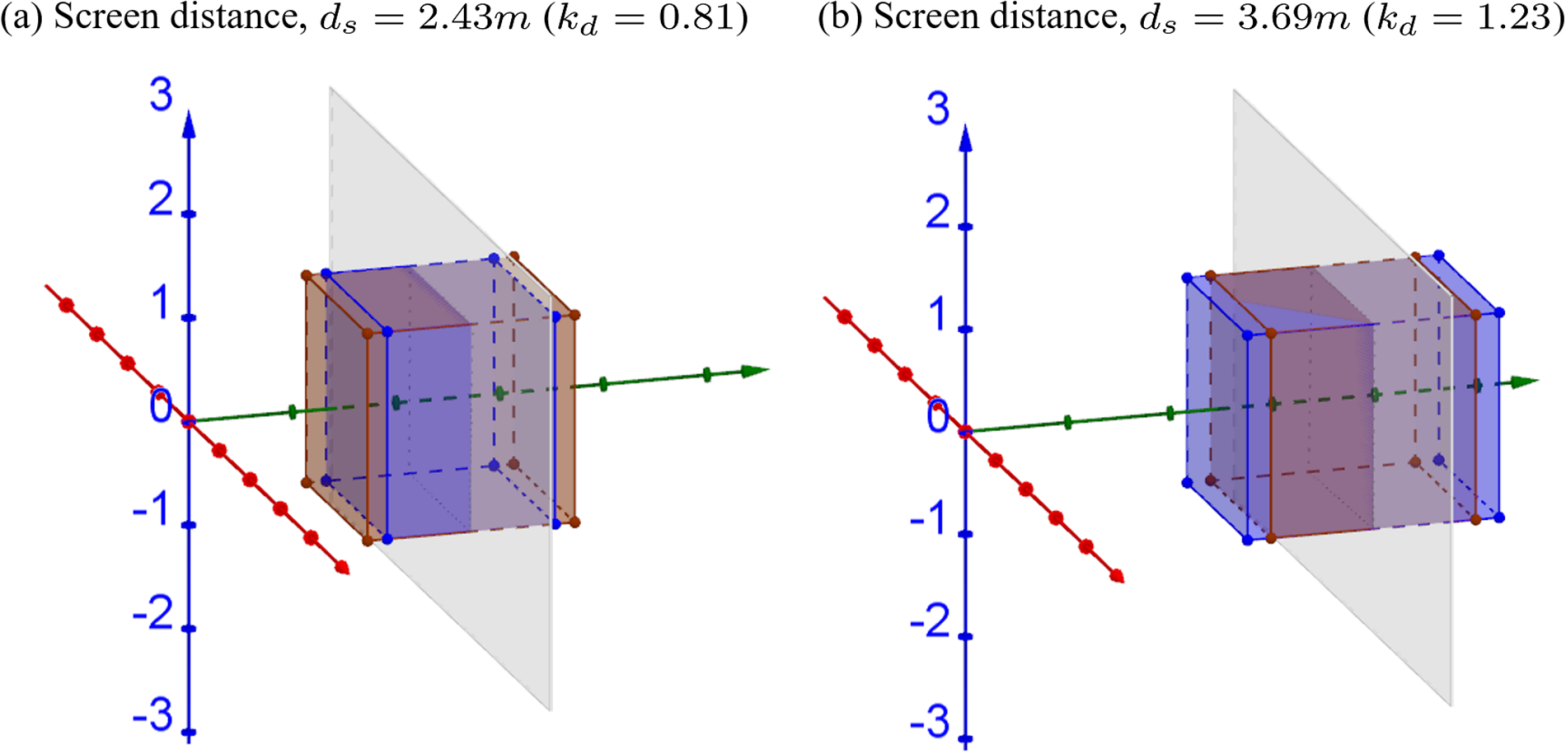
3D simulations of changing screen distance. The convergence distance is assumed to be fixed (*d*_*c*_ = 3*m*), while the screen distance to be (a) smaller, *d*_*s*_ = 2.43*m* (b) larger, *d*_*s*_ = 3.69*m* than the convergence distance. The brown cube is an orthoscopic representation of the 2*m* × 2*m* × 2*m* cube located at [0, 0, 3*m*]^⊺^ in the original world. The gray plane represents the display screen.

Fig 9 shows the 3D simulations when convergence and screen distances are mismatched. When the screen is closer than the convergence distance (*d*_*s*_ = 2.43*m*, *k*_*d*_ = 2^−0.3^ = 0.81 < 1), both front and rear surfaces of the cube appear compressed towards the screen, as shown in Fig 9a. When the screen is farther than the convergence distance (*d*_*s*_ = 3.69*m*, *k*_*d*_ = 2^0.3^ = 1.23 > 1), the cube appears expanded away from the screen, as shown in Fig 9b. The simulations confirm that changing the screen distance only affects the depth of the cube (i.e., in *z*-dimension).

**Fig 10:**
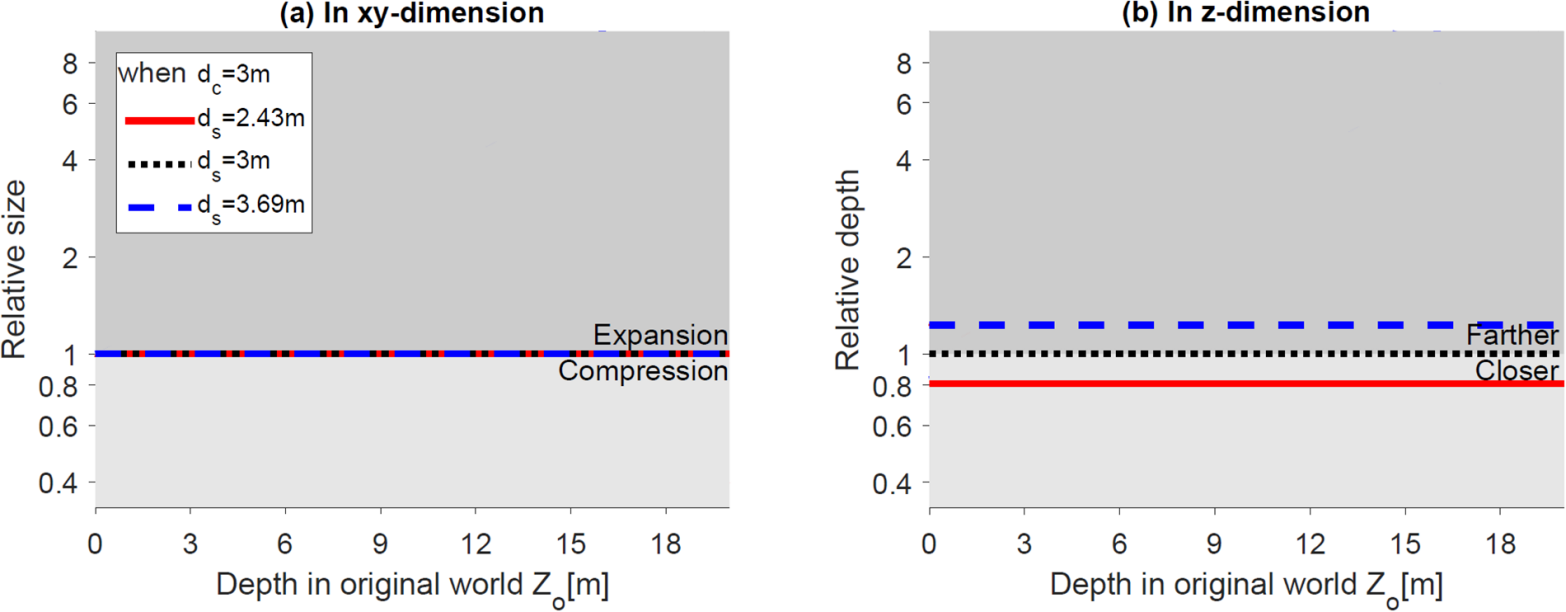
Effect of changing screen distance. (a) and (b) show the relative size (in *xy*-dimension) and depth (in *z*-dimension) of the reconstructed world along the *z*-axis, respectively. The horizontal black lines (relative size = 1 or depth = 1) represent no geometric distortion in the reconstructed world.

Fig 10 shows that the relative size and depth change as a function of the depth in the real world. The relative size in *xy*-dimension and relative depth in *z*-dimension can be expressed as $\frac{X_{p}}{X_{o}}=\frac{Y_{p}}{Y_{o}}=1$ and $\frac{Z_{p}}{Z_{o}}=k_{d}$, respectively. When screen distance is closer than (red solid lines) or farther than convergence distance (blue dashed lines), the relative size does not change, suggesting the linear size is independent of the screen distance (Fig 10a). In terms of depth, when screen distance is closer (red solid lines) or farther (blue dashed lines) than the convergence distance, the constructed world appears closer and compressed or farther and expanded, respectively (Fig 10b).

### Effect of changing camera convergence distance

This analysis assumes that only camera convergence distance is varying at given screen distance (*d*_*s*_ = 3*m*) and other parameter pairs are matched (*k*_*s*_ = 1, *k*_*f*_ = 1, *T* = [0, 0, 0]^⊺^). Since $k_{w}=\frac{w_{s}}{w_{c}}=\frac{2\;\text{tan}\left(\alpha_{sh}/2\right)d_{s}}{2\;\text{tan}\left(\alpha_{ch}/2\right)d_{c}}=k_{f}k_{d}$ and $k_{d}=\frac{d_{s}}{d_{c}}$, the transfer function (1) can be simplified as follows:

$$P=[\begin{array}{c} X_{p}\\ Y_{p}\\ Z_{p} \end{array}]=\frac{d_{s}}{Z_{o}\left(1-k_{d}\right)+d_{s}}[\begin{array}{c} X_{o}\\ Y_{o}\\ Z_{o} \end{array}].$$ (6)

**Fig 11:**
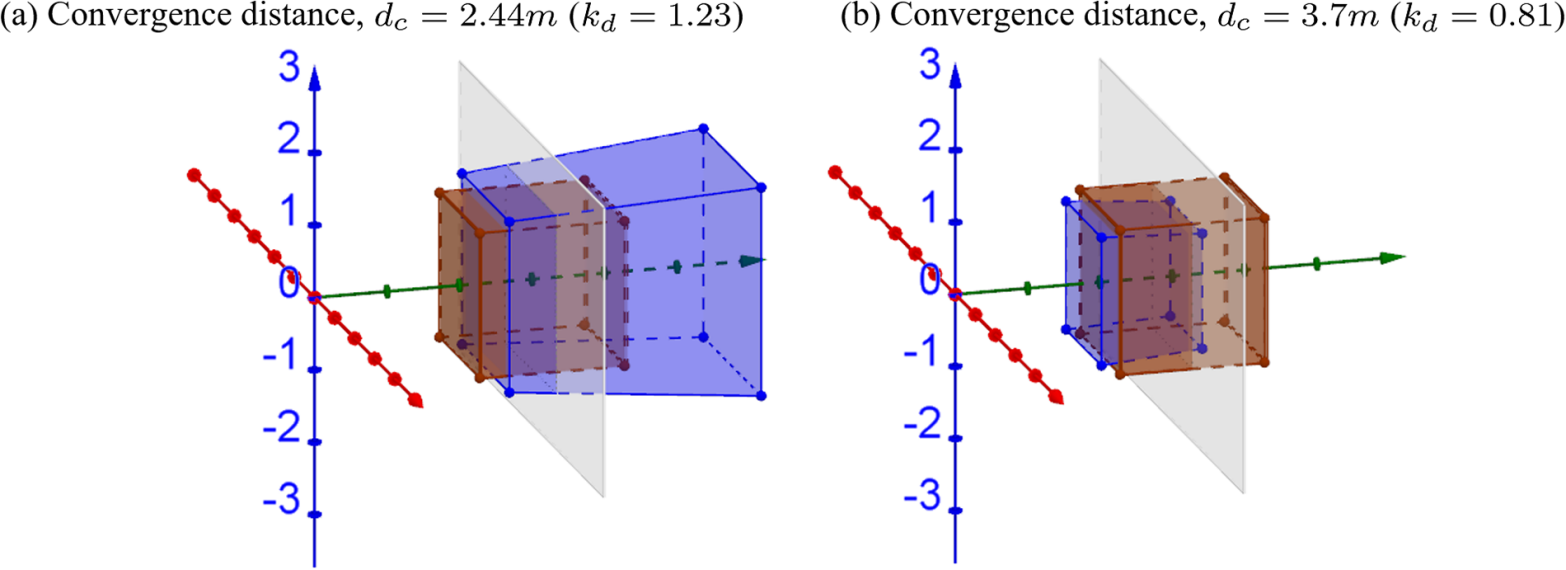
3D simulations of changing convergence distance. The screen distance is assumed to be fixed at *d*_*s*_ = 3*m*, while the camera convergence distance is (a) smaller *d*_*c*_ = 2.44*m* and (b) larger *d*_*c*_ = 3.7*m* than the screen distance. The brown cube and gray plane are the same as in Fig 5.

Fig 11 shows the 3D simulations when convergence and screen distances are mismatched. When camera convergence distance is smaller than screen distance (*d*_*c*_ = 2.44*m*), the reconstructed cube appears pushed father and larger (Fig 11a). When convergence distance is larger than screen distance (*d*_*c*_ = 3.7*m*), the reconstructed cube appears smaller and closer (Fig 11b). In both cases, more expansion/compression occurs at a farther distance.

**Fig 12:**
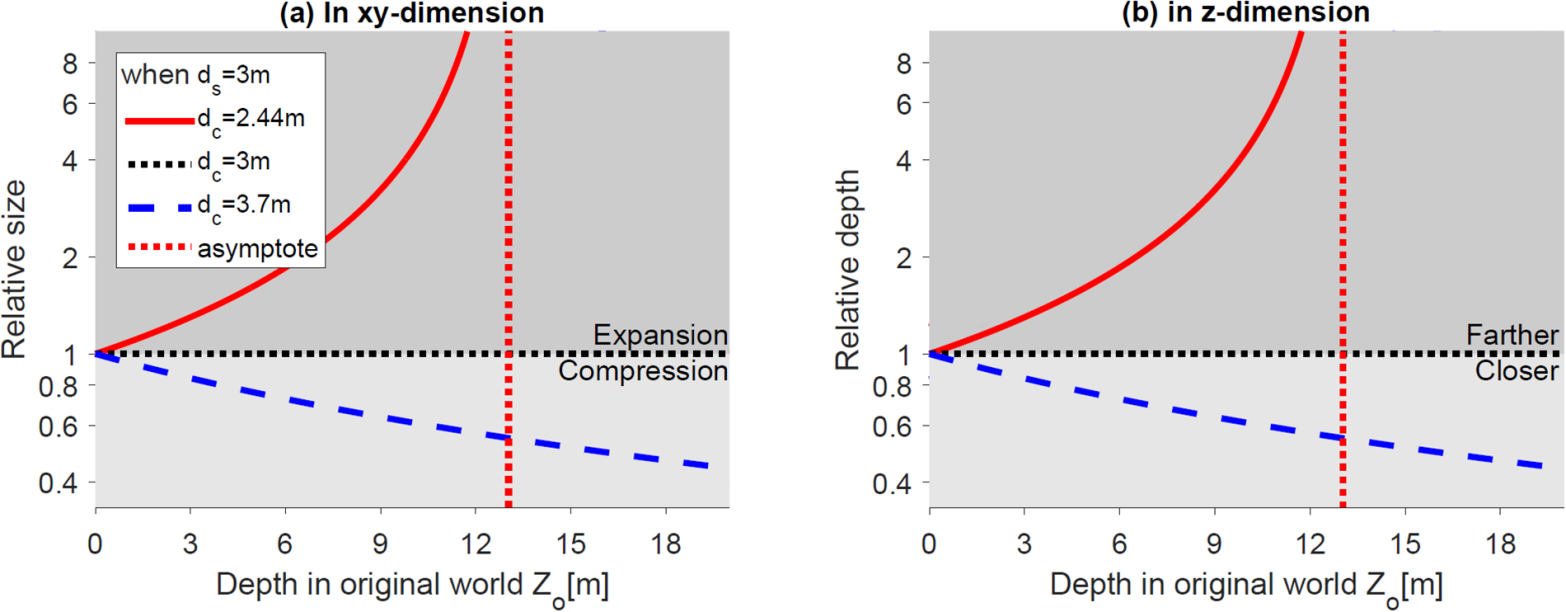
Effects of changing convergence distance. (a) and (b) show the relative size (in *xy*-dimension) and depth (in *z*-dimension), respectively of the reconstructed world along the *z*-axis. The red dotted vertical lines are the asymptotes of the red curves. The horizontal black lines (relative size = 1 or depth = 1) represent no geometric distortion in the reconstructed world.

Fig 12 shows the relative size and depth change compared to the orthoscopic condition. When camera convergence distance is shorter or larger than screen distance, the size of the object appears expanded (red solid line) or compressed (blue dashed line), respectively (Fig 12a). In terms of depth, objects appear farther (red solid line) or closer (blue dashed line) to the viewer, respectively (Fig 12b). The red dotted lines are the asymptotes of the red curves. When camera convergence distance is smaller than screen distance, the viewer may not be able to fuse objects farther than the asymptote and may see double vision.

The amount of geometric distortions (both size and depth ratio between the reconstructed object to original world object) monotonically increases as the distance from the viewer increases. When the amount of compression progressively increases along the depth direction, objects become thinner (in depth direction). Generally, the effect is more severe for distant objects. A distant object appears to be flat demonstrating the cardboard effect [10, 12] (Fig 12b).

Since objects in the foreground and background (i.e., different depths) are scaled in different ratios, the viewer will perceive objects as a miniaturization (i.e., the puppet theater effect [10, 11]) or enlargement effect. The mismatch between screen and camera convergence-distance results in a perceptual distortion called the *Alice in Wonderland syndrome* [17]. Observers with such syndrome experience various size and depth distortions such as micropsia (objects are perceived to be smaller than they actually are), macropsia (objects are perceived to be bigger than they actually are), peliopsia (objects are perceived to be closer than they actually are), and teliopsia (objects are perceived to be farther than they actually are).

An extreme case is worth further discussion where the convergence distance is infinity, i.e., the cameras are placed in parallel and without adjusting the convergence distance. In this case, the reconstructed world fits the following transfer function:

$$P=[\begin{array}{c} X_{p}\\ Y_{p}\\ Z_{p} \end{array}]=\underset{d_{c}\to \infty }{\text{lim}}\frac{d_{s}}{Z_{o}\left(1-\frac{d_{s}}{d_{c}}\right)+d_{s}}[\begin{array}{c} X_{o}\\ Y_{o}\\ Z_{o} \end{array}]=\frac{d_{s}}{Z_{o}+d_{s}}[\begin{array}{c} X_{o}\\ Y_{o}\\ Z_{o} \end{array}].$$ (7)

**Fig 13:**
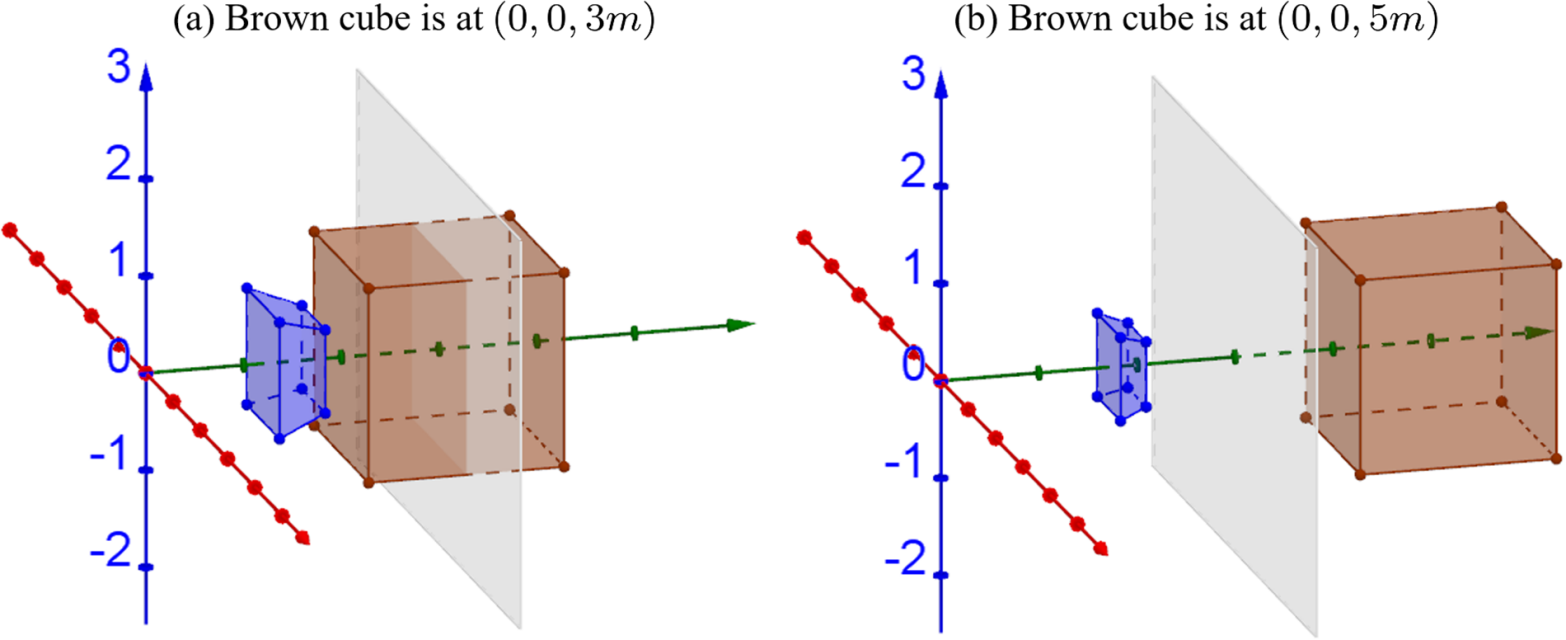
Extreme cases of camera convergence distance and screen distance mismatch, where the camera convergence distance is infinity (i.e., the cameras are parallel) and screen distance is, *d*_*s*_ = 3*m*. The cube in the original world is (a) located at [0, 0, 3*m*]^⊺^ and (b) located at [0, 0, 5*m*]^⊺^ The gray plane represents the display screen.

Fig 13 shows the 3D simulations when screen distance is *d*_*c*_ = 3*m*, where the cube in the original world is located at [0, 0, 3*m*]^⊺^ (Fig 13a) and [0, 0, 5*m*]^⊺^ (Fig 13b). In both cases, the apparent objects are in front of the screen (all in crossed disparity) and become smaller and closer. The compression of the depth is severer in farther cube condition (Fig 13b) because the depth at all distances (including infinite distance) is compressed in between the screen and viewer distance. As a result, the cardboard effect becomes amplified for distant objects.

### Distortion-free scaled reproduction

In Eq (1), if the three ratios between screen width to camera frustum width (*k*_*w*_), screen distance to camera convergence distance (*k*_*d*_), and eye separation to camera separation (*k*_*s*_) are the same (*k*_*w*_ = *k*_*d*_ = *k*_*s*_), and without head position offset, the three ratios can be denoted as *k* and the transfer function (1) can be simplified as follows:

$$P={\left[X_{p},Y_{p},Z_{p}\right]}^{⊺}=k{\left[X_{o},Y_{o},Z_{o}\right]}^{⊺}.$$ (8)

**Fig 14:**
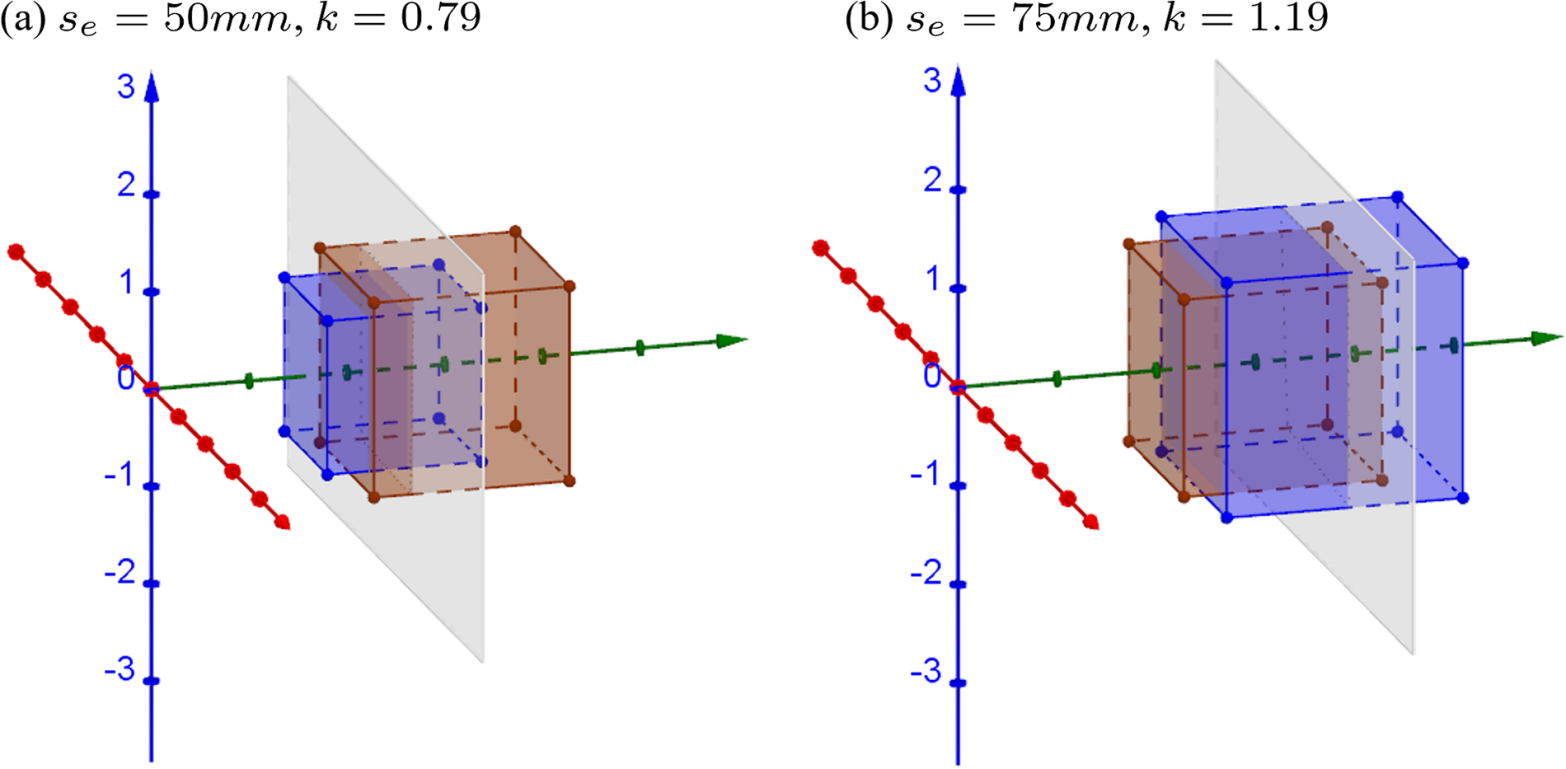
3D simulations of distortion-free reproduction. The camera separation is *s*_*c*_ = 63*mm* and convergence distance is assumed to be *d*_*c*_ = 3*m*. (a) The ratio between eye separation and camera separation is smaller than 1, (*s*_*e*_ = 50*mm*, *k*_*s*_ = 0.79) and other ratios are the same (*k*_*w*_ = *k*_*d*_ = *k*_*s*_ = 0.79). (b) The ratio between eye separation and camera separation is larger than 1, (*s*_*e*_ = 75*mm*, *k*_*s*_ = 1.19) and other ratios are the same (*k*_*w*_ = *k*_*d*_ = *k*_*s*_ = 1.19). The brown cube and gray plane are the same as in Fig 5.

In this case, *xyz* dimensions are scaled in the same ratio in different depths so that the reconstructed world is an undistorted but scaled version of the original world. Fig 14 shows examples of the 3D simulations when the ratio *k* is smaller (*k* = 0.79) and larger (*k* = 1.19) than orthoscopic condition (*k* = 1). When the ratio is smaller than 1, the cube appears smaller and closer, as shown in Fig 14a. When the ratio is larger than 1, the cube appears larger and farther, as shown in Fig 14b. However, the reproduced objects shape appears to be remained as a cube as it is presented in the original world.

**Fig 15:**
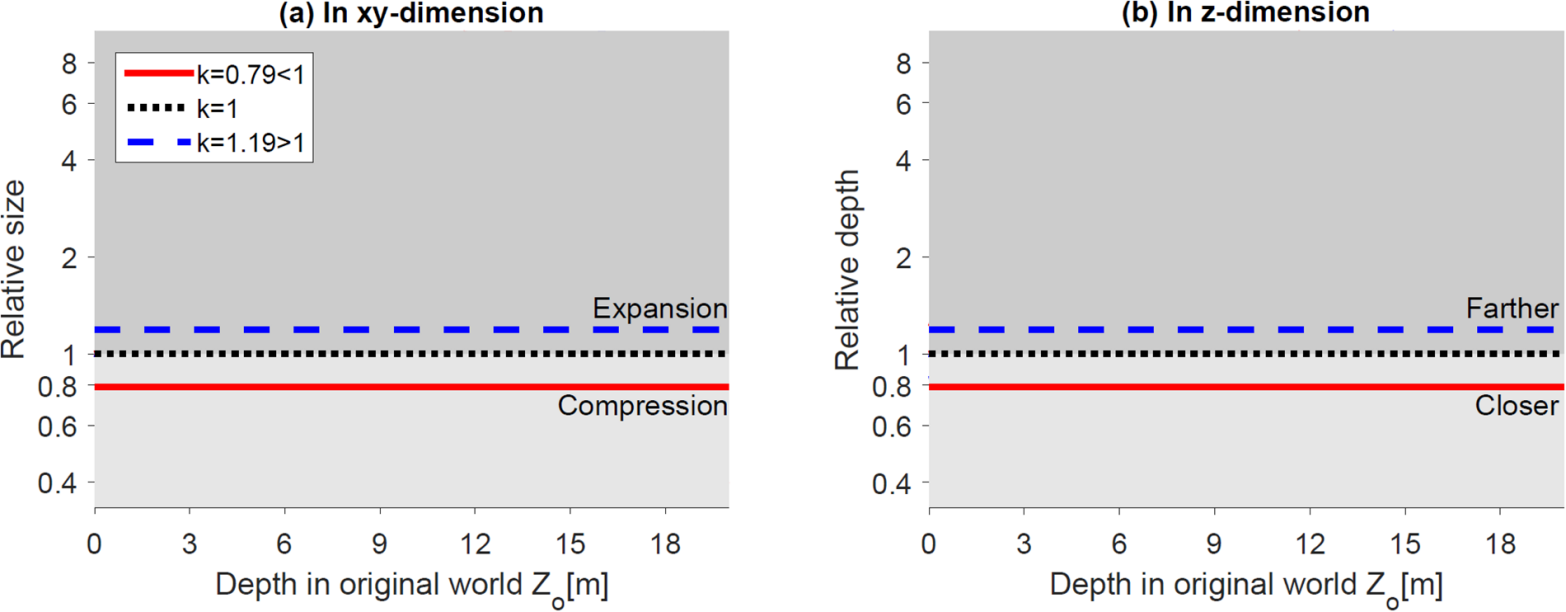
Distortion-free scaled reproduction. (a) and (b) show the relative size (in the *xy*-dimension) and depth (in the *z*-dimension) of the reconstructed world along the *z*-axis, respectively. The horizontal black lines (relative size = 1 or depth = 1) represent no geometric distortion in the reconstructed world.

Fig 15 shows that the relative size and depth change as a function of the depth in the real world. The relative size in *xy*-dimension can be expressed as $\frac{X_{p}}{X_{o}}=\frac{Y_{p}}{Y_{o}}=k$ and the relative depth in *z*-dimension can be expressed as $\frac{Z_{p}}{Z_{o}}=k$. When the ratio is smaller (red solid lines) or larger (blue dashed lines) than 1, both the relative size and depth are scaled in the same ratio (Fig 15a and 15b, respectively).

Note that since the reconstructed world is only scaled but not distorted in this condition, it provides a way to remove geometric distortions in S3D by adjusting the variables to equate the ratio of pairs.

### Effect of head translations

This analysis assumes that the three paired parameters are matched (*k*_*s*_ = 1, *k*_*w*_ = 1, *k*_*d*_ = 1). In this condition, the transfer function (1) is simplified as follows:

$$P=[\begin{array}{c} X_{p}\\ Y_{p}\\ Z_{p} \end{array}]=[\begin{array}{c} X_{o}\\ Y_{o}\\ Z_{o} \end{array}]+\frac{d_{c}-Z_{o}}{d_{c}}[\begin{array}{c} T_{x}\\ T_{y}\\ T_{z} \end{array}].$$ (9)

**Fig 16:**
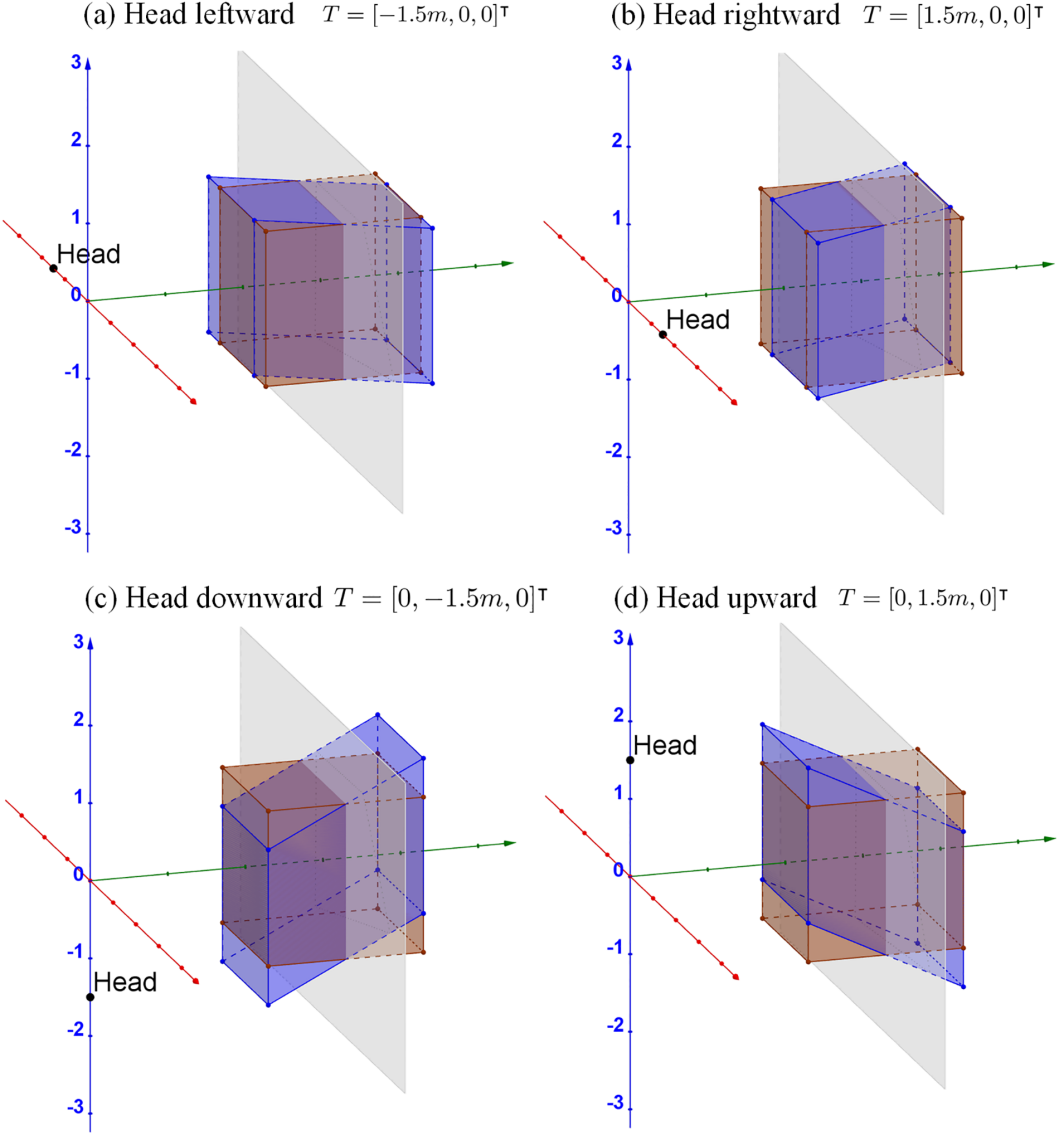
3D simulations of head translations. The convergence distance is assumed to be *d*_*c*_ = 3*m*. The viewer’s head is assumed to translate, as indicated by arrows (a) leftward, (b) rightward, (c) downward, and (d) upward, 1.5*m* from the optimal viewing position (origin). When the viewer’s head translates backward or forward, the distortions are the same as changing the screen distance farther or closer, respectively, as shown in Fig 9. The brown cube and gray plane are the same as in Fig 5.

Fig 16 shows examples of the 3D simulations when the viewer’s head translates from the idea position, corresponding to the position between the two cameras. When the viewer’s head translates to the left *T*_*x*_ = −1.5*m* or to the right *T*_*x*_ = 1.5*m*, the cubes are sheared to the left (Fig 16a) and right (Fig 16b), respectively. Similarly, when the viewer’s head translates downward *T*_*y*_ = −1.5*m* or upward *T*_*y*_ = 1.5*m*, the cubes are sheared downward (Fig 16c) and upward (Fig 16d), respectively. When the viewer’s head translates backward or forward, the distortion is the same as moving the screen farther and closer as discussed in section ‘*Effect of changing screen distance*’. The cubes are expanded away from the screen (Fig 9b) or compressed towards the screen (Fig 9a), respectively.

Overall, the part of the displayed cube in front of the screen moves in the same direction as the head translation, and the part behind the screen moves to the opposite direction of the head translations. Onscreen points stay on the screen without any change. Thus, the cube always appears to follow the head movements while maintain the fronto-parallel characteristics of the front and back surfaces. When the viewer’s head translates laterally (i.e., leftward, rightward, downward, and upward), our model indicates shearing distortions towards the viewer position. The distortion is apparent especially while the viewer is in motion. The backward or forward movements of the viewer’s head are basically the same as changing the screen distance farther or closer. Therefore, the consequent distortion patterns are analyzed in section ‘*Effect of changing screen distance*’.

## Guidelines for S3D Imaging content development

The results of our analyses suggest guidelines that may eliminate or minimize geometric distortion for content developers and users. These are explicitly developed below.

### Avoid large screen disparity

As analyzed above, when the ratio of eye separation to camera separation is larger than the ratio of screen width to camera frustum width at convergence distance (*k*_*s*_ > *k*_*w*_), the reconstructed world becomes more compressed (both in size and depth) at a larger depth (see blue curve in Fig 6 and red line in Fig 10). In these conditions, the cardboard effect may affect distant objects. In contrast, when the ratio of eye separation to camera separation is smaller than the ratio of screen width to camera frustum width at convergence distance (*k*_*s*_ < *k*_*w*_), the reconstructed world is more expanded at larger depths (see red curve in Fig 6 and blue line in Fig 10. In these conditions, the effect is opposite to the cardboard effect, we call it the expansion effect.

**Fig 17:**
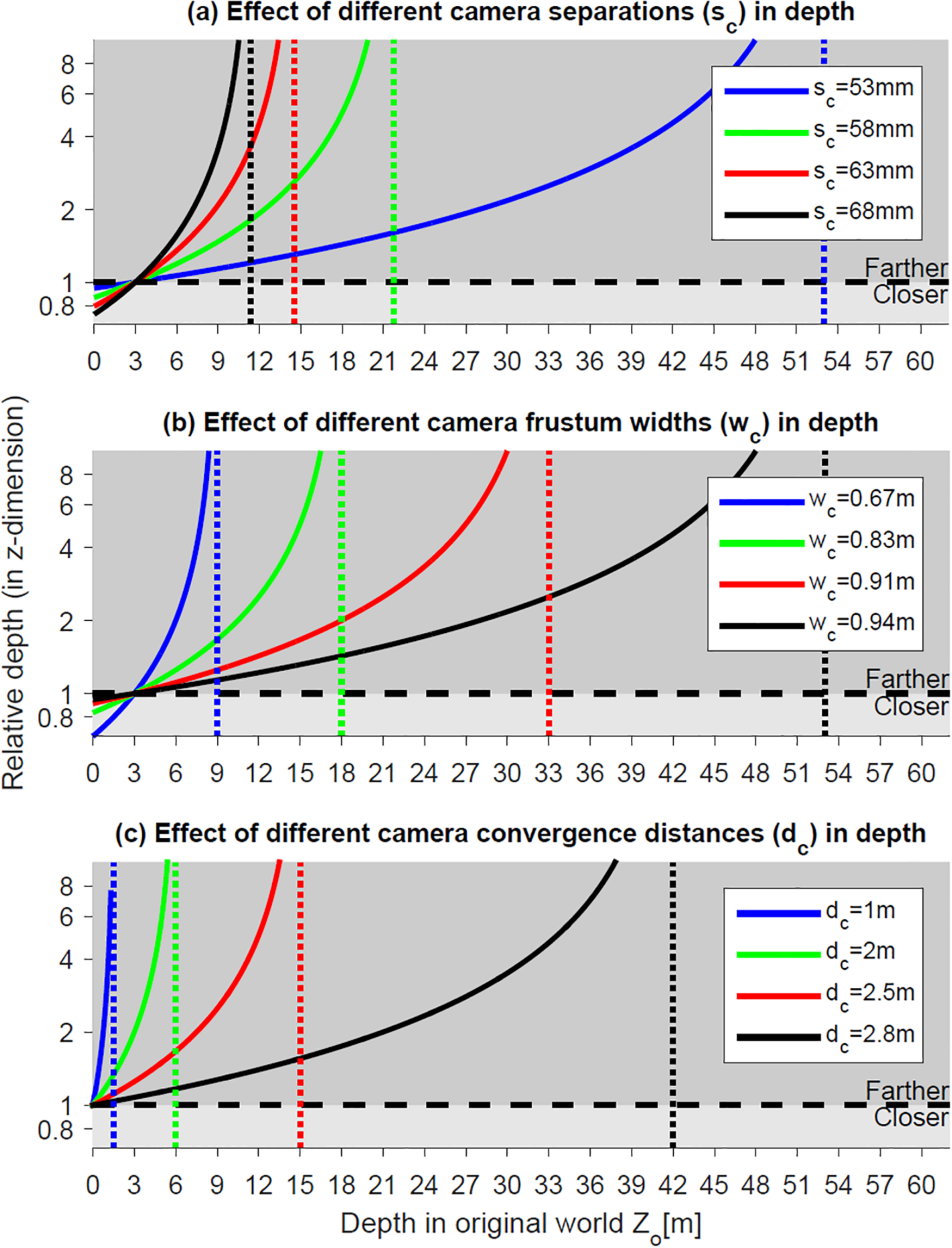
Distortion of the relative depth in the reconstructed world when *k*_*s*_ < *k*_*w*_ for (a) different camera separations (*s*_*c*_ = 53*mm*, 58*mm*, 63*m*, and 68*mm*), when eye separation and camera convergence distance are fixed (*s*_*c*_ = 50*mm* and *d*_*c*_ = 3*m*), (b) different camera frustum widths, i.e., for different camera FOVs (*w*_*c*_ = 0.67*m*, 0.83*m*, 0.91*m*, and 0.94*m*), when the screen size and convergence distance are fixed (*w*_*s*_ = 1*m* and *d*_*c*_ = 3*m*), (c) different camera convergence distances (*d*_*c*_ = 1*m*, 2*m*, 2.5*m*, and 2.8*m*), when screen distance is fixed (*d*_*s*_ = 3*m*). The solid curves represent relative depth distortions in the *z*-dimension as a function of the depth in original world. The dotted vertical lines are the asymptotes of the curves. The horizontal black dashed line (relative depth = 1) represents the orthoscopic condition without any geometric distortion.

More critically, the depth in the real world has asymptotic limits (i.e., when *k*_*s*_ < *k*_*w*_, $Z_{o}<\frac{k_{w}d_{c}}{k_{w}-k_{s}}$). Objects at depths farther than these limits are presented with large uncrossed screen disparities that the viewer may not be able to fuse, even if they are fixated. When eye separation is smaller than camera separation (*k*_*s*_ < *k*_*w*_ = 1), a smaller camera separation yields a larger asymptotic limit, as shown in Fig 17a. In addition, when only screen width is larger than camera frustum width (1 = *k*_*s*_ < *k*_*w*_), a larger camera FOV or camera convergence distance (i.e., a larger camera frustum width) yields a larger asymptotic limit, as shown in Fig 17b and 17c. Therefore, for S3D producers, a smaller camera separation, a larger camera convergence distance, or a larger camera FOV is recommended, so that $k_{s}=\frac{s_{e}}{s_{c}↓}\geq \frac{d_{s}\;\text{tan}\left(\alpha_{sh}/2\right)}{d_{c}↑\;\text{tan}\left(\alpha_{ch}↑/2\right)}=k_{w}$ to avoid large uncrossed screen disparities.

**Fig 18:**
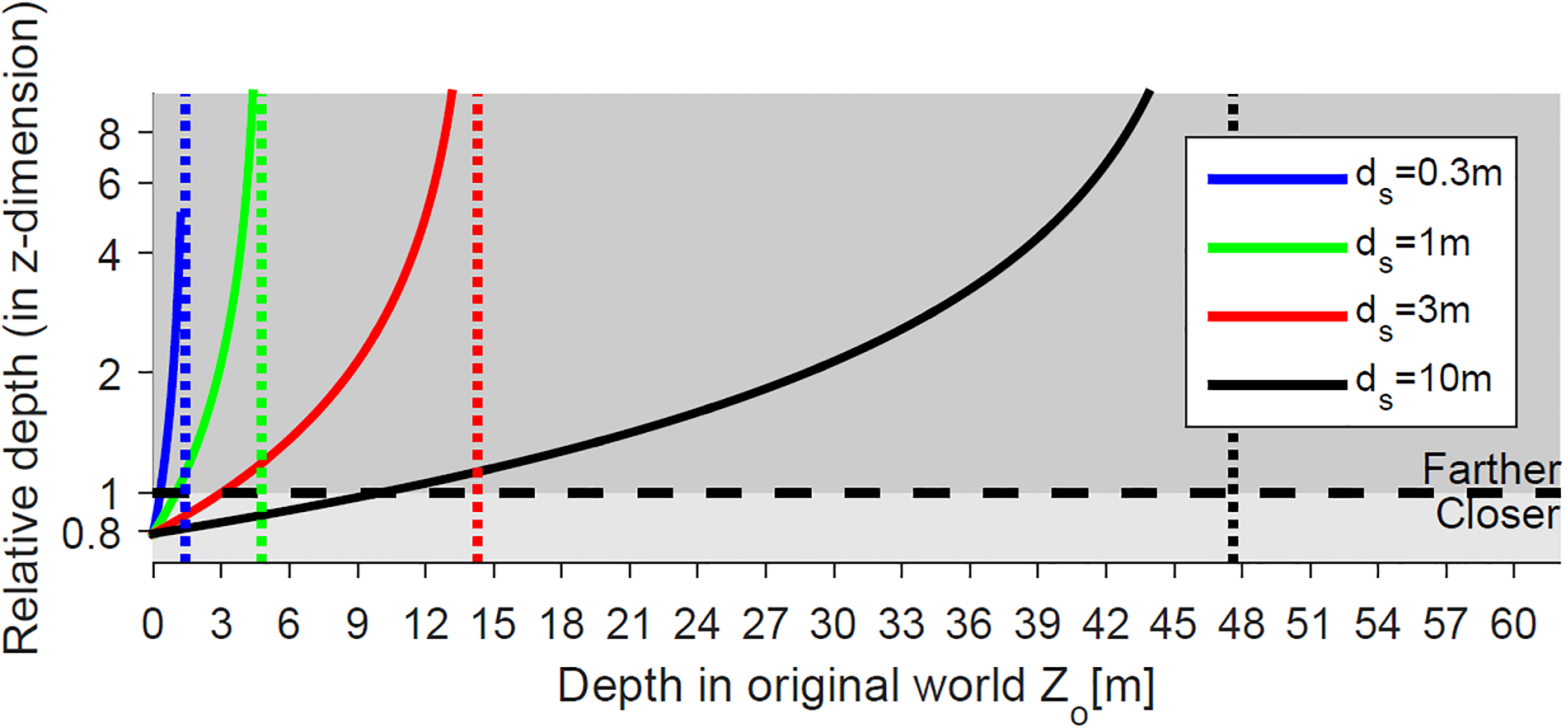
Distortion of the relative depth in the reconstructed world when *k*_*s*_ < *k*_*w*_ = 1 for different screen distances (*d*_*s*_ = 0.3*m*, 1*m*, 3*m* and 10*m*), when eye separation, camera separation, and convergence distance are fixed (*s*_*e*_ = 50*mm*, *s*_*c*_ = 63*mm*, and *d*_*c*_ = 3*m*). The solid curves, dotted lines, and black dashed line refer to Fig 17.

In following examples, we assume that camera convergence distance is set to be the same as the screen distance (*d*_*c*_ = *d*_*s*_) and consider four different screen distance options; *d*_*s*_ = 0.3*m* (mobile phone/tablet viewing distance), *d*_*s*_ = 1*m* (desktop monitor viewing distance), *d*_*s*_ = 3*m* (TV screen viewing distance), and *d*_*s*_ = 10*m* (movie theater screen viewing distance). Fig 18 shows the relative depth of the four viewing conditions when eye separation is smaller than camera separation (e.g., *s*_*e*_ = 50*mm* and *s*_*c*_ = 63*mm*, where *k*_*s*_ < *k*_*w*_ = 1). The four dotted vertical lines in Fig 18 are the asymptotic limits corresponding to the four convergence distance conditions. When camera convergence distance is the same as screen distance (*k*_*d*_ = 1), a larger screen distance results in a larger fusible limit on the original world distance.

When the depth composition in the original world have an asymptotic limit (i.e., *k*_*s*_ < *k*_*w*_), it is not desirable to model objects at depths farther than the asymptotic limit ($Z_{o}=\frac{k_{w}d_{c}}{k_{w}-k_{s}}$). For the S3D graphic rendering of the virtual world, the far plane of virtual camera frustum can be defined at or slightly beyond the asymptotic limit. Any objects farther than the far plane (e.g., mountains, clouds, or buildings) can be projected on the far plane as a 2D image (texture), which will make them appear at an infinite distance. Limiting the original virtual world to the asymptotic depth not only reduces the render time but also avoids the problem of large uncrossed screen disparity. For example, as shown in Fig 18, the asymptotic limit of the red curve (*s*_*e*_ = 50*mm*, *s*_*c*_ = 63*mm*, and *d*_*c*_ = 3*m*) is 14.3*m*. We define the far plane of camera frustum at 14.3*m* and project objects farther than the distance on the far plane as a 2D image so that objects at distances larger than 14.3*m* in the original world are perceived at an infinite distance.

### Correct geometric distortions

As discussed in section ‘*Distortion-free scaled reproduction*’, under many conditions it may be possible to eliminate geometric distortions in S3D by matching the ratios among the parameters pairs (instead of individually matching all the paired parameters). Under these conditions, the reconstructed world is only scaled from the original world but without distortions (Fig 14).

To equate the three ratios, we need to match screen FOV with camera FOV by adjusting screen distance, and match the distance ratio with the separation ratio by adjusting converge distance (i.e., *α*_*sh*_ = *α*_*ch*_ and $\frac{s_{e}}{s_{c}}=\frac{d_{s}}{d_{c}}$, resulting in *k*_*w*_ = *k*_*d*_ = *k*_*s*_). Users can adjust the screen distance by moving closer or farther from the screen, and adjust camera convergence distance by shifting the left and right view horizontally (e.g., increasing/decreasing convergence in NVIDIA 3D Vision [18] and ‘3D depth slider’ in Nintendo 3DS [19]). When screen distance is adjusted first, distortions from FOV mismatch will be eliminated (turn into a combination of Figs 5 and 11) and then the distortions of size scaling at different depths will be removed by adjusting convergence distance (turn into Fig 14). When convergence distance is adjusted first, distortions of size scaling at different depths will be eliminated (turn into Fig 9) and then the distortions of depth compression or expansion will be eliminated (turn into Fig 14) by adjusting screen distance.

More interesting (and possibly more intriguing) approach will be that we can combine different distortion patterns to compensate for each other. In real-world viewing condition, eye separation is fixed for each individual viewer and camera separation is usually set during the production. Our model guides us to correct the distortions caused by the mismatch of eye separation and camera separation. For example, when eye separation is smaller than camera separation (*s*_*e*_ < *s*_*c*_), farther distance objects appear larger and farther (Fig 5a). If this kind of distortion is combined with a distortion where convergence distance is larger than screen distance (*d*_*c*_ < *d*_*s*_)(Fig 11b), the various geometric distortions will compensate for each other. This compensation can result in a distortion-free (up to a scaling) reproduction of the original world depth structure (i.e., the case *α*_*sh*_ = *α*_*ch*_ and $\frac{s_{e}}{s_{c}}=\frac{d_{s}}{d_{c}}$). Delivering a scale but undistorted 3D structure may be sufficient for conveying the scene information [3]. Note that the ability of mix-and-match of available parameters to control various distortion is particularly important because, in many cases, S3D content production and consumption are two independent processes, where the production side cannot control the consumer’s display condition, leaving only limited control for the consumers since the parameters in the production process have already been set.

In some cases, the ability to adjust screen distance is constrained. For instance, the distance from the viewer to the TV cannot be larger than the length of a living room, or laptops cannot be too close to the viewer since it will be difficult for the viewer to focus. In such situations, size distortions (in *xy*-dimension) can be corrected by adjusting the convergence distance (i.e., making *k*_*w*_ = *k*_*s*_). However, an incorrect screen distance causes a mismatch between camera FOV and screen FOV, therefore, depths in the reconstructed world may be compressed or expanded. Such depth distortions can be eliminated by scaling the onscreen images so that the displayed images’ FOV is the same as camera FOV. For example, when the distance from the viewer to the TV cannot be larger than the length of a living room, one can scale down the onscreen images and only use part of the screen. When laptops cannot be too close to the viewer, one can scale up and display with only part of the images on the screen.

To eliminate the geometric distortions caused by the head translations, the viewing’s head needs to stay in front of the screen center (image center) or the viewer’s head position needs to be tracked, and then corresponding parameter adjustments should be applied to so that the reconstructed world is not sheared.

## Discussion

It should be obvious that our geometrical model of the S3D imaging and other models in the literature [2, 4] are fundamentally identical since they all veridically represent the capture and display processes. The advantage of our model is in its format that supports a more intuitive understanding of the relations between the various parameters and their impacts on geometric distortions. In S3D capture and display processes, various mismatches and distortions may combine. Our model, as presented in the transfer function (1), provides an intuitive tool for understanding the impact of each parameter mismatch on the distortion and their possible interactions. This isolated knowledge on the cause-effect with respect to the distortion pattern suggests us to a useful, but possibly trivial conclusion, that in order to eliminate the geometric distortions, all mismatches should be minimized. Specifically, for applications where the exact size of the scene may be important (e.g., teleoperation), it may be necessary to achieve an orthoscopic projection (i.e., *k*_*s*_ = 1, *k*_*w*_ = 1, and *k*_*d*_ = 1). In most other applications, distortion elimination with simple scale change (which is what we have proposed here) is likely to be acceptable.

Masaoka et al. [9] and Yamanoue [10] focused on the effects of camera separation and camera FOV. Their models had no explicit pairing of display screen distance and camera convergence distances. The mismatch of convergence distance and screen distance will affect the analysis of distortions caused by camera separation or FOV mismatches. For example, Yamanoue et al. [10] concluded that parallel-cameras configuration does not produce the puppet-theater effect. This is because the left and right images were horizontally shifted to the left and right, respectively, by a distance equivalent to half of the viewer’s IPD after the images were scaled to screen size (i.e., shifting images $\frac{d_{s}\;\text{tan}\left(\alpha_{sh}/2\right)}{d_{c}\;\text{tan}\left(\alpha_{sh}/2\right)}s_{c}/2=s_{e}/2$, see Eq (14) with scaling of $\frac{\text{tan}(\alpha_{sh}/2)}{\text{tan}(\alpha_{ch}/2)}$ in Appendix). Thus, the ratio of screen width to camera frustum width at convergence distance is the same as the ratio of eye separation to camera separation ($\frac{w_{s}}{w_{c}}=\frac{s_{e}}{s_{c}}$), resulting in the condition *k*_*s*_ = *k*_*w*_ in (1). Therefore, in the model of Yamanoue et al. [10], the sizes of the reconstructed objects are scaled by the same ratio at all depths.

Since the puppet-theater effect is defined as the size distortion between objects in the foreground and in the background, global magnification/minification of size does not induce the puppet-theater effect. However, this particular case does not cover the parallel cameras in all possible configurations. The parallel-cameras configuration still can cause the puppet-theater effect. The same method was used in [2] by Held and Banks when they analyzed the mismatch between camera separation and eye separation (see Fig 1(A) and 1(I) in the Appendix of [2] and compare to our results in Fig 5). In their modeling, the left and right images were also horizontally shifted to the left and right by the distance of half the viewer’s IPD, respectively. The convergence distance is also changed when changing camera separation in this case. Thus, the analysis of camera separation mismatch in [2] was confounded by the screen distance mismatch, which may be unclear to readers.

To avoid the issue of large uncrossed disparity on screens, a smaller camera separation, or a larger camera convergence distance or FOV (i.e., larger camera frustum width at convergence distance) are recommended for S3D content producers so that *k*_*s*_ > *k*_*w*_. For example, considering viewers have IPDs around 64*mm* and are expected to watch 50-inch TV at 3*m* screen distance (i.e., 41° screen FOV), if the camera convergence is also 3*m*, large screen disparities can be avoided by setting camera separation narrower than the expected viewers’, e.g., 60*mm* and camera FOV wider, e.g., 60° (giving *k*_*s*_ = 1.07 > *k*_*w*_ = 0.5). When *k*_*s*_ < *k*_*w*_ and asymptotic limit exists for the depth, we recommend that the far plane is defined at or slightly beyond the asymptotic limit and objects farther than the plane are projected on the plane as a 2D image (texture). Even though distant objects are perceived at an infinite distance in binocular stereo vision, monocular cues of the distant objects (e.g., farther mountains are occluded by closer mountains and have lighter colors, farther buildings are smaller than closer buildings) may be strong enough and users may not notice the difference from the original world.

As mentioned, the perception in a distorted S3D world is similar to the Alice in Wonderland syndrome [17], where the depth and size perception can be altered such that objects appear too close, too far, too big, or too small. For example, normal movements may appear too slow in a compressed space and too fast in an expanded space. Since the perception of motion within such a distorted space may lead to a perceptual inconsistency of the user’s egocentric motion expectations learned by real-world experiences, it may induce visually induced motion sickness (VIMS) [20, 21]. Thus, the perceptual inconsistency in a distorted space may be a likely source of VIMS in S3D [3].

The proposed geometric model can predict geometric distortions caused by the mismatches among image capture, display, and viewing, while perceptual distortions may not match and are usually smaller than the geometric distortions predicted by ray-intersection models [22, 23]. Geometric distortions predicted by ray-intersection models are solely determined by the binocular depth cue (binocular disparity). However, depth perception in 3D space involves both monocular and binocular depth cues. Human visual systems interpret depth by combining different depth cues [24–26]. Geometric distortions simulated in this paper are illustrated from a third-person perspective, but the viewer only sees the distortions from the first-person perspective (i.e., the origin in Figs 5, 7, 9, 11, 13 and 14, and head positions in Fig 16). Monocular depth cues (i.e., linear perspective, occlusion, shading, etc.) from the first-person perspective are largely unaffected by geometric distortions [27, 28]. Thus, monocular depth cues can effectively reduce and limit the effects of the size and depth distortions. However, the unaffected monocular depth cues are conflicting with the binocular depth cue in a distorted S3D space. Moreover, when the viewer’s head is translating laterally, motion parallax [29] (one of monocular depth cues) that exists in real life is missing since S3D displays can only provide the view (perspective) captured by the cameras. Head translations result in a strong perception of objects following the viewer’s movements. This depth cue conflict (intra-visual conflict) between monocular and binocular and the conflict between the absence of motion parallax and self-motion may cause VIMS [3].

We only discussed real screen displays (e.g., smartphone, monitor, TV, and movie theater), and not virtual screens displays (e.g., head mounted displays). There are two main differences between screen displays and head mounted displays. First, when adjusting screen distance in real screen displays, the screen FOV varies since the screen size is fixed. However, in head-mounted displays, when adjusting virtual screen distance by changing the lens-to-display distance, the virtual screen size varies while the virtual screen angular FOV remains fixed [30]. Second, in real screen displays, the camera separation is usually fixed in current 3D video games and movies. On the other hand, in head-mounted displays, users may be able to adjust the camera separation by changing the lens separation of the headset (e.g., Ocular Rift [31]). Therefore, in our discussion, camera separation was fixed and screen size was constant in the analysis of changing screen distance. However, there is no technical reason why the camera separation may not be under user control (at least over a restricted range in real screen applications).

The currently proposed geometric model has some limitations. We assume no viewer’s head rotations relative to the screen. This assumption does hold if the viewer sees S3D imagery in head-mounted displays, or the viewer’s head stays upright relative to the screen. However, the viewer’s head rotations with respect to the screen cause additional geometric distortions in the reconstructed S3D world [2]. We also assumed that camera image plane and screen image plane are parallel. However, in some cases, the image planes between image capture, display may be mismatched. As pointed by [2], yaw rotation (vertical-axis), roll rotation(forward-axis), and stereo images captured by convergence-axis but displayed on a flat screen will introduce vertical disparity, which may cause other problems (e.g., eye strain) for S3D viewing. These cases are outside the scope of the current paper. We are expanding our model to cover viewers’ head rotations and display image plane mismatches in a follow-up study.

## Appendix

In the derivation of the transfer function between the original world and the reconstructed world in S3D, simple pinhole cameras are assumed to be used for stereo scene capture (Fig 4a). The sensor-shift was modeled by relocating the image centers to the display center (Fig 4b). These aligned stereo images are assumed to be projected on a flat screen at a given screen distance, *d*_*s*_, then the intersection of the lines connecting left and right eye to the corresponding onscreen points are assumed to be the reconstructed point in S3D (Fig 4c).

The detailed derivation starts from the parallel-cameras capture with sensor shift technique in Fig 4a. The amount of the shifting determines the convergence distance, *d*_*c*_. The left and right cameras are at *C*_*l*_ = [−*s*_*c*_/2, 0, 0]^⊺^ and *C*_*r*_ = [−*s*_*c*_/2, 0, 0]^⊺^. The lines from *C*_*l*_ and *C*_*r*_ to a point in the original world, *O* = [*X*_*0*_, *Y*_*0*_, *Z*_*0*_]^⊺^, can be expressed as

$$l_{C_{l}O}=[-s_{c}/2,0,0{]}^{⊺}+\lambda_{1}[X_{o}+s_{c}/2,Y_{o},Z_{o}{]}^{⊺},$$ (10)

$$l_{C_{r}O}=[s_{c}/2,0,0{]}^{⊺}+\lambda_{2}[X_{o}-s_{c}/2,Y_{o},Z_{o}{]}^{⊺},$$ (11)

where λ_1_ and λ_2_ are line-equation parameters. Since the captured images will be presented on the screen to the viewer, we derived the distance from the pinhole apertures to the camera sensors as the screen distance. The image plane at the screen distance can be expressed as *ip*_*s*_: *z* = *d*_*s*_. The intersections of the two lines, $l_{C_{l}O}$ and $l_{C_{r}O}$ with the image plane can be obtained by equating the *z* components of the line equations and the image plane (i.e. 0 + λ_1_*Z*_*o*_ = *d*_*s*_ and 0 + λ_2_*Z*_*o*_ = *d*_*s*_). After rearranging the equations, we get λ_1_ = λ_2_ = *d*_*s*_/*Z*_*o*_. By substituting the line-equation parameters with *d*_*s*_/*Z*_*o*_, the two points *S*_*l*1_ and *S*_*r*1_ can be expressed as

$$S_{l1}=[\frac{d_{s}\left(X_{o}+s_{c}/2\right)}{Z_{o}}-s_{c}/2,\frac{d_{s}Y_{o}}{Z_{o}},d_{s}{]}^{⊺},$$ (12)

$$S_{r1}=[\frac{d_{s}\left(X_{o}-s_{c}/2\right)}{Z_{o}}+s_{c}/2,\frac{d_{s}Y_{o}}{Z_{o}},d_{s}{]}^{⊺}.$$ (13)

In Fig 4a, the centers of the captured images are misaligned. When the captured images are displayed on a single screen, the centers of the captured images are aligned at the screen center automatically. The displacement of the image centers can be calculated from the two blue similar triangles in Fig 4b,

$$\frac{\text{Amount}\;\text{of}\;\text{displacement}}{s_{c}/2}=\frac{d_{c}-d_{s}}{d_{c}}\Rightarrow \text{Amount}\;\text{of}\;\text{displacement}=\frac{d_{c}-d_{s}}{d_{c}}s_{c}/2,$$ (14)

which is $\frac{d_{c}-d_{s}}{d_{c}}s_{c}/2-s_{c}/2=-\frac{d_{s}}{d_{c}}s_{c}/2$ in terms of the captured image centers. After adjusting the displacement, the points *S*_*l*2_ and *S*_*r*2_ can be expressed as

$$S_{l2}=[\frac{d_{s}\left(X_{o}+s_{c}/2\right)}{Z_{o}}-\frac{d_{s}}{d_{c}}s_{c}/2,\frac{d_{s}Y_{o}}{Z_{o}},d_{s}{]}^{⊺},$$ (15)

$$S_{r2}=[\frac{d_{s}\left(X_{o}-s_{c}/2\right)}{Z_{o}}+\frac{d_{s}}{d_{c}}s_{c}/2,\frac{d_{s}Y_{o}}{Z_{o}},d_{s}{]}^{⊺}.$$ (16)

When the captured images are displayed on a screen with *α*_*sh*_, angular FOV while the camera FOV is *α*_*ch*_, the size of the captured images (i.e., the *x* and *y* components of the Eqs (15) and (16)) should be scaled by $\frac{\text{tan}(\alpha_{sh}/2)}{\text{tan}(\alpha_{ch}/2)}$. The resulting onscreen positions of the *S*_*l*_ and *S*_*r*_ are

$$S_{l}=[\frac{\text{tan}(\alpha_{ch}/2)}{\text{tan}(\alpha_{sh}/2)}\left(\frac{d_{s}\left(X_{o}+s_{c}/2\right)}{Z_{o}}-\frac{d_{s}}{d_{c}}s_{c}/2\right),\frac{\text{tan}(\alpha_{ch}/2)}{\text{tan}(\alpha_{sh}/2)}\frac{d_{s}Y_{o}}{Z_{o}},d_{s}{]}^{⊺},$$ (17)

$$S_{r}=[\frac{\text{tan}(\alpha_{ch}/2)}{\text{tan}(\alpha_{sh}/2)}\left(\frac{d_{s}\left(X_{o}-s_{c}/2\right)}{Z_{o}}+\frac{d_{s}}{d_{c}}s_{c}/2\right),\frac{\text{tan}(\alpha_{ch}/2)}{\text{tan}(\alpha_{sh}/2)}\frac{d_{s}Y_{o}}{Z_{o}},d_{s}{]}^{⊺}.$$ (18)

Now, we need to reconstruct the 3D structure from the positions of two onscreen points and the viewer’s two eyes. Note that in our geometric model, we assumed that a point with onscreen horizontal disparity is virtually reconstructed at the intersection of the two lines passing each eye and the corresponding onscreen point. Given the two onscreen points, (17) and (18), and two eyes positions, *E*_*l*_ = [−*s*_*e*_/2, 0, 0]^⊺^ + [*T*_*x*_, *T*_*y*_, *T*_*z*_]^⊺^ = [− *s*_*e*_/2 + *T*_*x*_, *T*_*y*_, *T*_*z*_]^⊺^ and *E*_*r*_ = [*s*_*e*_/2, 0, 0]^⊺^ + [*T*_*x*_, *T*_*y*_, *T*_*z*_]^⊺^ = [*s*_*e*_/2 + *T*_*x*_, *T*_*y*_, *T*_*z*_]^⊺^, the projection lines passing the left and right eyes to the corresponding onscreen points can be expressed as

$$l_{E_{l}P_{l}}\;=[\begin{array}{c} -s_{e}/2+T_{x}\\ T_{y}\\ T_{z} \end{array}]\;+\;\lambda_{3}[\begin{array}{c} \frac{\text{tan}(\alpha_{ch}/2)}{\text{tan}(\alpha_{sh}/2)}\left(\frac{d_{s}\left(X_{o}\;+\;s_{c}/2\right)}{Z_{o}}\;-\;\frac{d_{s}}{d_{c}}s_{c}/2\right)+s_{e}/2-T_{x}\\ \frac{\text{tan}(\alpha_{ch}/2)}{\text{tan}(\alpha_{sh}/2)}\frac{d_{s}Y_{o}}{Z_{o}}-T_{y}\\ d_{s}-T_{z} \end{array}],$$ (19)

$$l_{E_{r}P_{r}}\;=[\begin{array}{c} s_{e}/2+T_{x}\\ T_{y}\\ T_{z} \end{array}]\;+\;\lambda_{4}[\begin{array}{c} \frac{\text{tan}(\alpha_{ch}/2)}{\text{tan}(\alpha_{sh}/2)}\left(\frac{d_{s}\left(X_{o}\;-\;s_{c}/2\right)}{Z_{o}}\;+\;\frac{d_{s}}{d_{c}}s_{c}/2\right)-s_{e}/2-T_{x}\\ \frac{\text{tan}(\alpha_{ch}/2)}{\text{tan}(\alpha_{sh}/2)}\frac{d_{s}Y_{o}}{Z_{o}}-T_{y}\\ d_{s}-T_{z} \end{array}],$$ (20)

where λ_3_ and λ_4_ are another set of line-equation parameters. The intersection of these two lines can be computed by equating (19) and (20). From the computation of *y* and *z* components (i.e., λ_3_ = λ_4_) and *x* component, i.e.,

$$\begin{array}{c} \;-s_{e}/2+T_{x}+\lambda_{3}\left[\frac{\text{tan}(\alpha_{ch}/2)}{\text{tan}(\alpha_{sh}/2)}\left(\frac{d_{s}\left(X_{o}+s_{c}/2\right)}{Z_{o}}-\frac{d_{s}}{d_{c}}s_{c}/2\right)+s_{e}/2-T_{x}\right]\\ =s_{e}/2+T_{x}+\lambda_{3}\left[\frac{\text{tan}(\alpha_{ch}/2)}{\text{tan}(\alpha_{sh}/2)}\left(\frac{d_{s}\left(X_{o}-s_{c}/2\right)}{Z_{o}}+\frac{d_{s}}{d_{c}}s_{c}/2\right)-s_{e}/2-T_{x}\right], \end{array}$$ (21)

we can find that $\lambda_{3}=\lambda_{4}=\frac{Z_{o}\frac{s_{e}}{s_{c}}}{Z_{o}\left(\frac{s_{e}}{s_{c}}-\frac{\text{tan}(\alpha_{ch}/2)}{\text{tan}(\alpha_{sh}/2)}\frac{d_{s}}{d_{c}}\right)+\frac{\text{tan}(\alpha_{ch}/2)}{\text{tan}(\alpha_{sh}/2)}d_{s}}$. Therefore, the virtually reconstructed point is located at

$$\begin{array}{cc} P=[\begin{array}{c} X_{p}\\ Y_{p}\\ Z_{p} \end{array}]= & \frac{\frac{\text{tan}(\alpha_{sh}/2)}{\text{tan}(\alpha_{ch}/2)}d_{s}\frac{s_{e}}{s_{c}}}{Z_{o}\left(\frac{s_{e}}{s_{c}}-\frac{\text{tan}(\alpha_{sh}/2)}{\text{tan}(\alpha_{ch}/2)}\frac{d_{s}}{d_{c}}\right)+\frac{\text{tan}(\alpha_{sh}/2)}{\text{tan}(\alpha_{ch}/2)}d_{s}}[\begin{array}{c} X_{o}\\ Y_{o}\\ Z_{o}\frac{\text{tan}(\alpha_{ch}/2)}{\text{tan}(\alpha_{sh}/2)} \end{array}]\\ + & \frac{\frac{\text{tan}(\alpha_{sh}/2)}{\text{tan}(\alpha_{ch}/2)}\frac{d_{s}}{d_{c}}\left(d_{c}-Z_{o}\right)}{Z_{o}\left(\frac{s_{e}}{s_{c}}-\frac{\text{tan}(\alpha_{sh}/2)}{\text{tan}(\alpha_{ch}/2)}\frac{d_{s}}{d_{c}}\right)+\frac{\text{tan}(\alpha_{sh}/2)}{\text{tan}(\alpha_{ch}/2)}d_{s}}[\begin{array}{c} T_{x}\\ T_{y}\\ T_{z}\frac{\text{tan}(\alpha_{ch}/2)}{\text{tan}(\alpha_{sh}/2)} \end{array}]. \end{array}$$ (22)

In (22), the parameters are shown in three pairs: the camera separation (*s*_*c*_) vs. eye separation (*s*_*e*_), the camera convergence distance (*d*_*c*_) vs. screen distance (*d*_*s*_), and the camera FOV (*α*_*ch*_) vs. screen FOV (*α*_*sh*_). If we substitute the comparable parameters with the ratios of the parameter pairs, i.e., $k_{s}=\frac{s_{e}}{s_{c}}$, $k_{d}=\frac{d_{s}}{d_{c}}$, and $k_{f}=\frac{\text{tan}(\alpha_{sh}/2)}{\text{tan}(\alpha_{ch}/2)}$, we can get a transfer function from the original world to the reconstructed world using the ratios of the corresponding parameter pairs:

$$P=[\begin{array}{c} X_{p}\\ Y_{p}\\ Z_{p} \end{array}]=\frac{k_{s}k_{f}k_{d}d_{c}}{Z_{o}\left(k_{s}-k_{f}k_{d}\right)+k_{f}k_{d}d_{c}}[\begin{array}{c} X_{o}\\ Y_{o}\\ Z_{o}/k_{f} \end{array}]+\frac{k_{f}k_{d}\left(d_{c}-Z_{o}\right)}{Z_{o}\left(k_{s}-k_{f}k_{d}\right)+k_{f}k_{d}d_{c}}[\begin{array}{c} T_{x}\\ T_{y}\\ T_{z}/k_{f} \end{array}].$$ (23)

The FOV ratio (*k*_*f*_) and distance ratio (*k*_*d*_) are independent for virtual screen displays (e.g., head mounted displays) where the screen distance can be adjusted by changing the power of screen lenses (in HMDs) while the screen FOV is not affected. However, more practically, for real screen displays (e.g., monitors and TVs), the screen size is usually fixed and the screen FOV changes when adjusting the screen distance. We rearrange the FOV and distance ratios as follows,

$$k_{f}k_{d}=\frac{\text{tan}\left(\alpha_{sh}/2\right)}{\text{tan}\left(\alpha_{ch}/2\right)}\frac{d_{s}}{d_{c}}=\frac{w_{s}}{w_{c}}=k_{w},$$ (24)

where *k*_*w*_ is the ratio of screen width (*w*_*s*_) to camera frustum width at convergence distance (*w*_*c*_) so that they are independent of screen distance *d*_*s*_. By replacing *k*_*f*_ *k*_*d*_ in (23), the transfer function can be expressed as follows:

$$P=\;[\;\begin{array}{c} X_{p}\\ Y_{p}\\ Z_{p} \end{array}\;]\;=\frac{k_{s}k_{w}d_{c}}{Z_{o}\left(k_{s}-k_{w}\right)+k_{w}d_{c}}\;[\;\begin{array}{c} X_{o}\\ Y_{o}\\ Z_{o}\frac{k_{d}}{k_{w}} \end{array}\;]\;+\frac{k_{w}\left(d_{c}-Z_{o}\right)}{Z_{o}\left(k_{s}-k_{w}\right)+k_{w}d_{c}}\;[\;\begin{array}{c} T_{x}\\ T_{y}\\ T_{z}\frac{k_{d}}{k_{w}} \end{array}\;]\;.$$ (25)

The transfer function here is controlled by camera convergence distance, *d*_*c*_, the three ratios (*k*_*s*_, *k*_*d*_, *k*_*w*_), and the offset of head position (*T*). Since the viewer cannot see objects located behind the viewer, *Z*_*p*_ (i.e., the depth of *P*) should be positive, thus, the coefficient has the constraint,

$$\frac{k_{s}k_{w}d_{c}}{Z_{o}\left(k_{s}-k_{w}\right)+k_{w}d_{c}}>0\Rightarrow Z_{o}<\frac{k_{w}d_{c}}{k_{w}-k_{s}}\left(\text{when}\;k_{s}<k_{w}\right).$$ (26)

Therefore, the depth in original world has an asymptotic limit when the ratio of eye separation to camera separation (*k*_*s*_) is smaller than the ratio of screen width to camera frustum width at convergence distance (*k*_*w*_). The limitation of the depth exists because the uncrossed disparity of two onscreen points should be smaller than the viewer’s IPD so that the two projection lines (from the two eyes to the two corresponding onscreen points) intersect in front of the viewer. The disparity of two onscreen points *D* can be expressed as

$$D=S_{rx}-S_{lx}=\frac{\text{tan}\left(\alpha_{sh}/2\right)}{\text{tan}\left(\alpha_{ch}/2\right)}\frac{d_{s}}{d_{c}}s_{c}\frac{Z_{o}-d_{c}}{Z_{o}}=k_{w}s_{c}\frac{Z_{o}-d_{c}}{Z_{o}},$$ (27)

which is also independent of the screen distance *d*_*s*_.
